# Identification of CDK7 Inhibitors from Natural Sources Using Pharmacoinformatics and Molecular Dynamics Simulations

**DOI:** 10.3390/biomedicines9091197

**Published:** 2021-09-10

**Authors:** Vikas Kumar, Shraddha Parate, Gunjan Thakur, Gihwan Lee, Hyeon-Su Ro, Yongseong Kim, Hong Ja Kim, Myeong Ok Kim, Keun Woo Lee

**Affiliations:** 1 Department of Bio & Medical Big Data (BK4 Program), Division of Life Sciences, Research Institute of Natural Science (RINS), Gyeongsang National University (GNU), 501 Jinju-daero, Jinju 52828, Korea; vikaspathania777@gmail.com; 2 Plant Molecular Biology and Biotechnology Research Center (PMBBRC), Division of Applied Life Science, Gyeongsang National University (GNU), 501 Jinju-daero, Jinju 52828, Korea; parateshraddha@gmail.com (S.P.); pika890131@gmail.com (G.L.); 3 Department of Veterinary Medicine, Institute of Animal Medicine, Gyeongsang National University (GNU), Jinju 52828, Korea; thakur.gunjan.123@gmail.com; 4 Department of Bio & Medical Big Data (BK4 Program), Research Institute of Life Sciences, Gyeongsang National University (GNU), Jinju 52828, Korea; rohyeon@gnu.ac.kr; 5 School of Cosmetics and Food Development, Kyungnam University, Masan 631-701, Korea; kimys@kyungnam.ac.kr; 6 Division of Life Sciences and Applied Life Science (BK21 Four), Research Institute of Natural Science (RINS), Gyeongsang National University (GNU), 501 Jinju-daero, Jinju 52828, Korea; hongjaac@gnu.ac.kr

**Keywords:** CDK7, cancer, pharmacophore, molecular docking, MD simulation, MM-PBSA, pharmacokinetic properties

## Abstract

The cyclin-dependent kinase 7 (CDK7) plays a crucial role in regulating the cell cycle and RNA polymerase-based transcription. Overexpression of this kinase is linked with various cancers in humans due to its dual involvement in cell development. Furthermore, emerging evidence has revealed that inhibiting CDK7 has anti-cancer effects, driving the development of novel and more cost-effective inhibitors with enhanced selectivity for CDK7 over other CDKs. In the present investigation, a pharmacophore-based approach was utilized to identify potential hit compounds against CDK7. The generated pharmacophore models were validated and used as 3D queries to screen 55,578 natural drug-like compounds. The obtained compounds were then subjected to molecular docking and molecular dynamics simulations to predict their binding mode with CDK7. The molecular dynamics simulation trajectories were subsequently used to calculate binding affinity, revealing four hits—ZINC20392430, SN00112175, SN00004718, and SN00262261—having a better binding affinity towards CDK7 than the reference inhibitors (CT7001 and THZ1). The binding mode analysis displayed hydrogen bond interactions with the hinge region residues Met94 and Glu95, DFG motif residue Asp155, ATP-binding site residues Thr96, Asp97, and Gln141, and quintessential residue outside the kinase domain, Cys312 of CDK7. The in silico selectivity of the hits was further checked by docking with CDK2, the close homolog structure of CDK7. Additionally, the detailed pharmacokinetic properties were predicted, revealing that our hits have better properties than established CDK7 inhibitors CT7001 and THZ1. Hence, we argue that proposed hits may be crucial against CDK7-related malignancies.

## 1. Introduction

Cancer is one of the leading causes of death in the 21st century and the most critical obstruction for the upsurge of the global lifespan [1]. As a result, the pharmaceutical industry and scientific communities have all focused on reducing the cancer-related death rate, with the expectation of rapid development of effective and safe cancer treatment. Generally, the genetic alteration in signaling pathways that control cell-cycle progression, apoptosis, and cell growth is the common hallmark of the disease progression [2]. Among the reasons mentioned above, the abnormal cell cycle is one of the most protruding features of tumor cells. Anomalous expression of cell-cycle-related proteins provides tumor cells their invasive, metastatic, drug-resistant, and anti-apoptotic properties [3]. The cell cycle is a highly regulated process managed by numerous checkpoints to safeguard the division and proliferation in an ideal way. The central machines that drive the cell-cycle progression are mediated by cyclin-dependent kinases (CDKs) and partner cyclins [4,5]. The CDKs are a family of approximately 20 serine/threonine kinases that regulate the fundamental processes [6]. CDKs are primarily divided into two main groups; the first ones are the cell-cycle linked CDKs (CDK1, 4, and 6) that directly regulate the cell-cycle progression, and the second ones are the transcription-linked CDKs (CDK7, 8, 9, 12, and 13) [7]. CDK7 is a unique member of the CDK family among transcription-associated kinases due to its dual function in cell-division control and transcription [8]. CDK7 forms a dimeric complex with MAT1, which is an element of various chromatin remodeling complexes. Furthermore, the dimeric complex with additional involvement of cyclin H is known as CDK-activating kinase (CAK), which phosphorylates the T-loop of corresponding CDK members (CDKs 1, 2, 4, and 6) to regulate the cell cycle [9,10]. The transcriptional regulation by CDK7 or CAK is performed by phosphorylating the carboxy-terminal domain (CTD) of RNA polymerase II at serine 5 and 7, as well as other transcription factors [11,12,13]. Bartkova et al., have identified the expression of CDK7 in normal and tumor cells for the first time [14]. According to recent studies, CDK7 overexpression is reported in various malignancies such as hepatocellular carcinoma, gastric cancer, oral squamous cell carcinoma, breast cancer, ovarian cancer, high-grade glioma, cholangiocarcinoma, pancreatic cancer, and colorectal cancer with aggressive clinicopathological features and poor prognosis [15,16,17,18,19,20,21,22,23,24,25]. As a result of CDK7’s direct involvement in numerous cancers, it has become an attractive target in cancer therapy [26,27,28]. To date, only one ATP-bound human CDK7 crystal structure is known [29]. The structure reveals that the ATP-binding site is located in the cleft between the residues of the N-terminal and C-terminal lobes. Interestingly, CDK7 has a 44% sequence similarity with CDK2 with a reported root mean square deviation of 1.25 Å [29]. The available structural and functional information of CDK7 was exploited previously by researchers to develop inhibitors that can bind to the ATP-binding site of CDK7 [28,30,31,32]. The literature survey confirms that great progress has been made over the past few years in discovering and developing CDK inhibitors during the last decade. Still, unfortunately, very few inhibitors were reported effective against CDK7 due to adverse effects and low efficacy [30,31]. The CDK7 inhibitors are classified either as reversible or irreversible. The first selective, reversible small-molecule inhibitor identified against CDK7 was BS-181, inhibiting CDK7 with a half-maximal inhibitory concentration (IC_50_) of 21 nM [33]. Interestingly, BS-181 was derived using computer-aided drug designing from roscovitine. Further studies based on BS-181 led to the identification of ICE0942 (CT7001) which inhibited CDK7 with an IC_50_ of 40 nM and is currently under Phase I clinical trials [30,34,35]. The inhibitors mentioned above have demonstrated nanomolar potency against CDK7 while inhibiting numerous other CDK family members, limiting their usage as selective inhibitors [31]. Intriguingly, Nathanael’s group identified THZ1, a phenylaminopyrimidine derivative, as the first irreversible ATP-competitive (covalent) inhibitor, inhibiting CDK7 with an IC_50_ of 3.2 nM and showing less affinity towards other CDK members [36]. To further improve the potency and selectivity of THZ1, Syros Pharmaceuticals developed SY-1365 which inhibits CDK7 with an IC_50_ of 84 nM and is currently under Phase I clinical trials [30]. Apart from small success in inhibiting CDK7, there is a continuous need for research in the design of selective inhibitors due to the dual role of CDK7 in cell-cycle regulation and transcription. A survey of literature reported numerous studies for inhibiting CDK7 considering in vitro or in silico strategies. However, a ligand- and structure-based pharmacophore study has not reported for CDK7 to date. Therefore, the present study was undertaken to explore the molecular mechanism of the selective binding of inhibitors with CDK7 over CDK2. In the current study, ligand- and structure-based pharmacophore models were generated to obtain the key features responsible for CDK7 inhibition. The generated pharmacophore models were validated and used as 3D queries to screen a drug-like database of natural compounds. The obtained compounds were subsequently subjected to molecular docking for the identification of the binding mode with CDK7. Furthermore, molecular dynamics simulations were performed on individual complexes to study the stability and binding affinity of the potential inhibitors with CDK7 under physiological conditions. The identified hit compounds were then checked for their in silico selectivity towards CDK7 over CDK2, using molecular docking studies. Finally, the detailed in silico pharmacokinetic properties were predicted for the identified hits, which may be helpful for their optimization or synthesis for further studies.

## 2. Materials and Methods

### 2.1. Ligand-Based Pharmacophore Generation

The ligand-based pharmacophore can be built as either a qualitative (common feature pharmacophore) or quantitative (3D-QSAR) model [37]. Owing to the lesser number of inhibitors reported against CDK7 to date, a reliable 3D-QSAR model cannot be generated. Therefore, in the present study, a ligand-based common feature pharmacophore approach was selected. In the literature, two types of inhibitors were reported for CDK7 inhibition, viz. selective or non-selective [28,30,31]. Rationally, a small set of well-known selective inhibitors was selected as a training set for hypothesis generation [31]. The training set compounds were downloaded from the PubChem database in SDF format and checked manually in *Discovery Studio* (DS) v18 (www.accelrys.com (accessed on 25 March 2021)) Accelrys Inc. San Diego, CA, USA). The compounds were then energy minimized using the *Steepest Descent* algorithm with CHARMM force field in DS. Before pharmacophore generation, the *Feature Mapping* module of the DS was used to identify the most common features of the training set compounds. The pharmacophore model generation was then carried out with the Common Feature Pharmacophore Generation module of DS. This module is based on the Hip-Hop algorithm, which identifies the three-dimensional (3D) spatial arrangements of chemical *features* common to training set molecules. A maximum of 255 hypothesis conformations were generated using the *BEST* algorithm with an energy threshold of 20 kcal/mol. Ten pharmacophore models were generated with various parameters such as the rank of the hypothesis, features, direct hit, partial hit, and max fit. During the hypothesis generation, special weightage was given to well-known CDK7 inhibitors—CT7001 and THZ1—by applying Principal and Max Omit feat values 2 and 0, respectively, to ensure that the inhibitor’s chemical features are considered in building pharmacophore space [38]. At the same time, other training set compounds were regarded as reasonably active, where all but one feature must map to the compound. 

### 2.2. Structure-Based Pharmacophore Generation

To build a reliable structure-based pharmacophore model, a protein’s 3D structural complex with a highly active ligand is a prerequisite. Lolli et al., reported the first X-ray crystal structure in 2004 for CDK7 in complex with ATP [29]. Thenceforth, no other ligand-bound X-ray crystal structure was reported with CDK7. Interestingly, electron microscopy (EM)-derived CDK7 structure, bound with the highly selective covalent inhibitor, THZ1, was deposited recently in Protein Data Bank (PDB) (PDB ID: 6XD3) [39]. The structure was downloaded and prepared in DS using the Clean Protein module. The unwanted molecules were removed, and the Receptor-Ligand Pharmacophore Generation module was used to generate the pharmacophore model. This module develops selective pharmacophore models based on protein–ligand interactions [40]. The *BEST* algorithm was opted for the conformation generation with the flexible fitting method, which generates ten hypotheses with different feature sets and selectivity scores. The best hypothesis was selected based on validation parameters and key interacting features with active site residues.

### 2.3. Validation of the Pharmacophore

Validation of the pharmacophore model is an essential step for its selection and evaluation. In the present study, two commonly used validation approaches, mainly, the receiver operating characteristic (ROC) curve and the Güner–Henry (GH) approach, were used [41,42]. The ROC curve analysis was performed during hypothesis generation in both ligand- and structure-based procedures. First, a small dataset was prepared with known active and inactive compounds. The four compounds used for pharmacophore generation were considered as known actives, and the other eight were taken as inactive. The top three hypotheses from each approach were selected and further validated with a second validation technique, the GH or decoy set method. A decoy set of 110 compounds was generated with 6 already known active inhibitors of CDK7 (IC_50_ < 100 nm) [30,31] and 104 inactive compounds. The Ligand Pharmacophore Mapping module in DS was used to screen the decoy dataset. The resulting mapping data were used for assessment of the pharmacophore quality by evaluating the following equation:

$$GF=(\frac{Ha}{4HtA})\;(3A+Ht)\times \{1-\frac{Ht-Ha}{D-A}\}$$

The selected and validated hypotheses from the ligand- and structure-based pharmacophore procedures were exploited as 3D queries to screen four natural compound databases in DS.

### 2.4. Drug-like Database Generation and Virtual Screening

Four natural compound libraries (ZINC, SuperNatural2, ExiMed, and InterBioScreen) were considered in the present study. To retrieve the drug-like small-molecule compounds from these libraries, it is recommended to filter the huge number of compounds during early steps to save time and unnecessary effort at a later stage in drug discovery [43,44]. Therefore, the libraries were filtered at first with Lipinski’s rule of five (Ro5) and then for their ADMET (absorption, distribution, metabolism, excretion, and toxicity) properties in DS [45,46]. The validated hypotheses from the ligand- and structure-based approaches were then used to map the compounds using the Ligand Pharmacophore Mapping protocol in DS, with the *FAST* algorithm for generating the conformations with the flexible fitting method. The obtained compounds were then checked manually aligning with the generated models, and the most appropriate ones were selected for the molecular docking study.

### 2.5. Molecular Docking

Molecular docking is a productive and cost-effective technique in computational drug design to identify and assess molecular interactions between the ligands and receptors [47]. In the present work, the aforementioned drug-like database constructed from natural sources was used for docking with the active site of CDK7 using the Genetic Optimization for Ligand Docking (GOLD v5.2.2) program [48]. The only available inhibitor-bound structure of CDK7 (PDB ID: 6XD3) was used for the molecular docking study. The active site for molecular docking was defined within a 13.10 Å radius around the THZ1, using DS’s Define and Edit Binding Site module. The docking sphere’s X, Y, and Z coordinates were 116.84, 95.12, and 79.66, respectively. In general, ten docking runs/ligand were performed for consensus generation. For the scoring of ligands, the commonly used scoring functionGoldScore fitnesswas used. The GoldScore fitness function is a molecular mechanics-like function optimized for predicting the ligand-binding site considering hydrogen bonding energy, van der Waals energy, metal interactions, and torsion deformations [49]. Higher GoldScore than reference (REF) inhibitors were used as a cut-off criterion for the selection of potential CDK7 binders. The compounds were further filtered based on their binding mode and vital residual interactions with active site residues reported as necessary for inhibiting CDK7 in the cell.

### 2.6. Molecular Dynamics Simulation

Molecular dynamics (MD) simulations were conducted using the Groningen Machine for Chemical Simulations (GROMACS v5.1.5) to understand better the molecular interaction mechanism of protein and potential hit chemicals under physiological settings in a better way [50]. The simulation parameters and coordinates of CDK7 and potential hit compounds were generated using CHARMM27 force field in GROMACS and SwissParam, respectively [51,52]. The TIP3P water model was used for hydration during the simulation run, and the prepared system was energy minimized with a force of 10 kJ/mol to avoid steric hindrance. The equilibration step was performed under NVT and NPT ensembles for 500 ps at 300 K, using a V-rescale thermostat and Parrinello–Rahman barostat, respectively [53,54]. All the simulations were run under periodic boundary conditions for 50 ns. The MD simulation trajectories were analyzed using DS and visual molecular dynamics (VMD) [55].

### 2.7. Binding Free Energy Calculations

The docking studies used to calculate the binding affinity for the target protein ignores the flexibility of the protein. Despite the advantage of screening large libraries of ligands in a short time, the docking scoring functions often lead to inconsistent results [56]. Therefore, a more reliable method, molecular mechanics Poisson−Boltzmann surface area (MM-PBSA), is generally used to rank the simulated complexes. The combined MD simulations and MM-PBSA calculations can incorporate the conformational fluctuations and entropic contributions to the binding energy [57]. The g_mmpbsa plugin tool in GROMACS was used to calculate the binding free energy from MD simulation trajectories [58]. The precise method used to calculate the binding free energy can be found elsewhere [59]. In general terms, the binding free energy of the protein–ligand complex in the solvent can be calculated as

$$\Delta G_{bind}=G_{complex}-(G_{protein}+G_{ligand})$$

where *G_complex_* is the total free energy of the protein–ligand complex whereas *G_protein_* and *G_ligand_* are the total free energies of the protein and ligand alone in the solvent, respectively. The final *ΔG_bind_* value for the CDK7–ligand complex was computed as the average value from the last 40 to 50 ns of the MD simulation trajectories.

### 2.8. In Silico Specificity over CDK2

Designing small-molecule inhibitors with selectivity profiles that will ultimately be successful in the clinic is a huge concern in kinase drug research due to higher similarities among the family members [60,61]. The literature survey reveals that CDK7 shares high similarities with one of its family members, CDK2 [29]. Therefore, to select a specific inhibitor for CDK7, we performed molecular docking of the selected hits from MD simulation analysis with CDK2. The crystal structure of CDK2 in complex with CT7001 was obtained from PDB (PDB ID: 5JQ5) [62]. As mentioned earlier, the structure was prepared in DS, and the GOLD program was used for molecular docking with similar docking parameters. The results were analyzed based on docking scores and fundamental residual interactions.

### 2.9. In Silico Prediction of Pharmacokinetic Properties

Predicting the pharmacokinetic properties (PK) using in silico tools is a common step in drug discovery to identify novel inhibitors [38,63,64]. The PK properties, including sub-categories in absorption, distribution, metabolism, excretion, and toxicity of a particular compound, were considered. The detailed in silico prediction of the PK properties might be helpful for further optimization of the selected hit as a successful leader. Therefore, in the present study, PK properties were predicted using an online webserver, *pkCSM* [65]. The selected hits were converted to their SMILES format in BIOVIA Draw and used as input for assessing their properties (http://biosig.unimelb.edu.au/pkcsm/ (accessed on 30 May 2021)). The output results were analyzed according to the threshold values and compared with REF inhibitors CT7001 and THZ1 used in the study. 

## 3. Results

**Figure 1:**
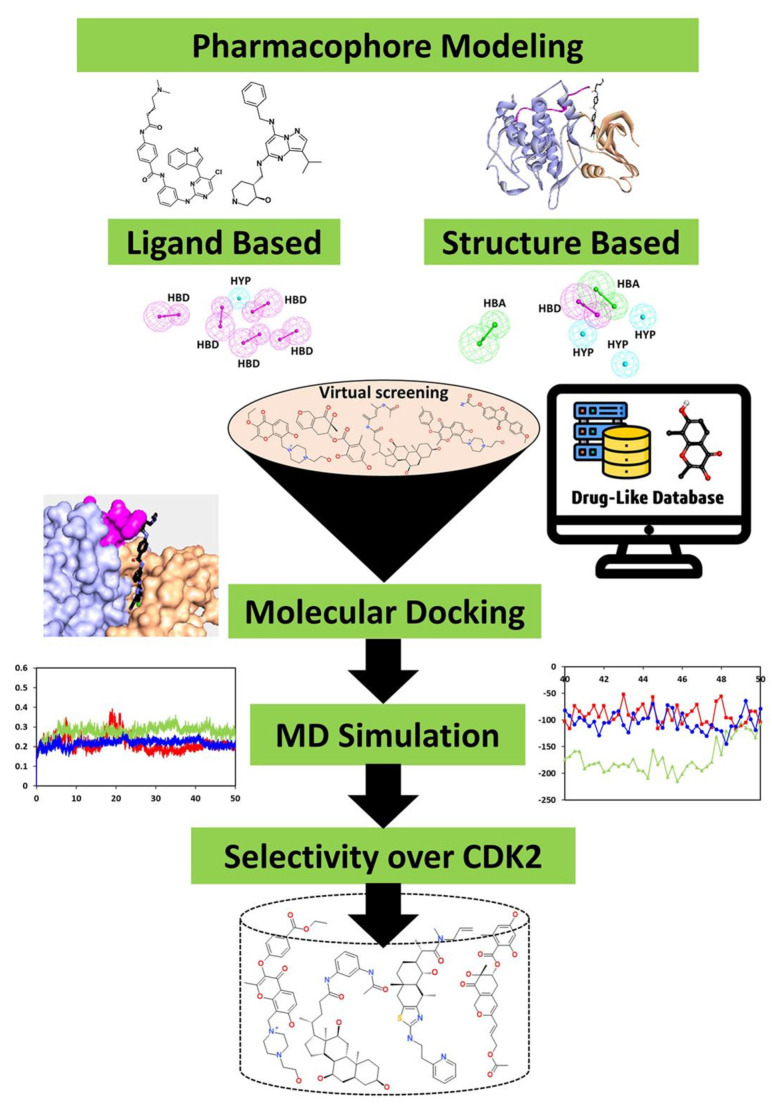
Schematic workflow for ligand and structure-based drug design used in the present study.

The identification of prospective and specific inhibitors of CDK7 was conducted in the present study using several computational methodologies. A general overview of the study is depicted (Figure 1).

### 3.1. Ligand-Based Pharmacophore Generation

**Figure 2:**
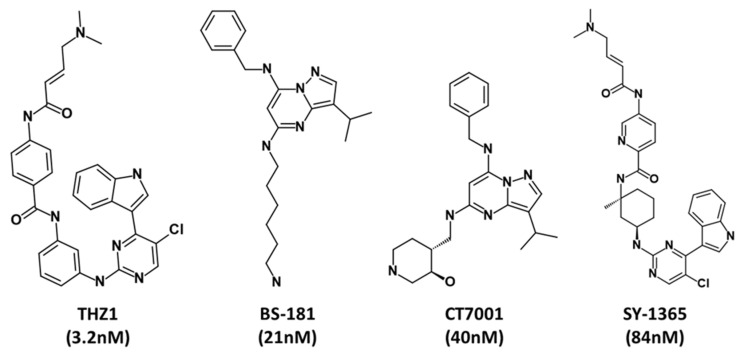
The two-dimensional structures of the compounds used as a training set for ligand-based common feature pharmacophore generation.

A qualitative common feature pharmacophore model was generated using a small group of selective inhibitors known against CDK7 (Figure 2). 

The Feature Mapping module in DS was used to select the most common features present in the training set compounds, which revealed a higher number of hydrogen bond donor (HBD), hydrogen bond acceptor (HBA), ring aromatic (RA), hydrophobic (HYP), and positive ionizable (PI) features. The obtained information was then used as input for the pharmacophore generation. A total of ten hypotheses (Hypo) were generated with a narrow range of ranking scores (67.71 to 69.73), indicating that all ten models’ features were spatially ordered. A deeper insight revealed that a minimum of 3–5 HBD features was present in all the hypotheses. A detailed overview of the generated hypothesis is given (Table 1). 

As mentioned in the Section 2, a validation step with ROC was performed simultaneously during the generation of the hypothesis. The ROC results revealed that Hypo1, Hypo2, and Hypo7 showed the highest specificity score of 0.75, 0.87, and 0.87, respectively (Table S1). The specificity score is the fraction of the genuinely inactive compounds being correctly recognized, and its value ranges from zero to one [66]. Therefore, the hypotheses mentioned above were selected for further analysis based on ROC analysis (Figure S1).

### 3.2. Structure-Based Pharmacophore Generation

**Figure 3:**
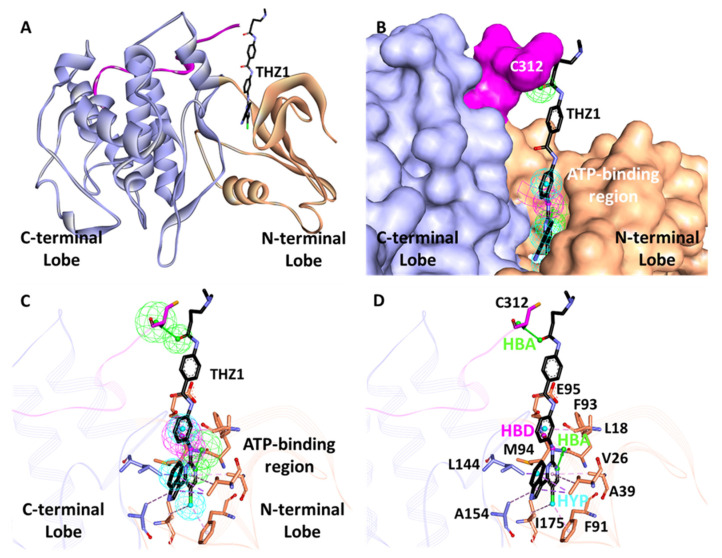
Generation of the structure-based pharmacophore. (**A**) CDK7 in complex with THZ1 was used to generate pharmacophore hypothesis. The N- and C-terminal of CDK7 are shown in tan and purple color, respectively. The region outside of the kinase domain is shown in pink. THZ1 is shown as a black stick. (**B**) The 3D representation of the pharmacophore inside the ATP-binding pocket of CDK7. (**C**,**D**) Mapping of pharmacophore hypothesis with the ATP-binding site residues and residue located outside the kinase domain Cys312 of CDK7. The surrounding residues are shown as sticks, and the hypothesis features are represented as hydrogen bond donor (HBD, magenta), hydrophobic (HYP, cyan), and hydrogen bond acceptor (HBA, green).

The CDK7 structure consists of an N- and C-terminal, which includes the kinase domain of the protein ranges from 12 to 295 amino acids (Figure 3A,B). The ATP-binding region of the protein primarily contains residues 90 to 170 (Figure 3C,D). 

The *Receptor-Ligand Pharmacophore Generation* protocol generated a total of ten hypotheses with five to six features. A minimum of three to four features was hydrophobic in all the hypotheses, and the selectivity score ranged from 8.79 to 10.31. A detailed summary of the hypothesis, such as the number and types of features, and selectivity score, is given (Table 2). The ROC analysis further revealed Hypo1, Hypo3, and Hypo4 displayed a specificity value of 0.75, 0.75, and 0.87 in terms of identifying inactive compounds; therefore, these hypotheses were initially selected for further study (Table S2 and Figure S1).

### 3.3. Pharmacophore Validation

Hypothesis validation is an essential step in pharmacophore modeling to check the performance of the hypothesis generated. The two commonly used approaches, ROC and GH, were used to validate the generated hypotheses [41,42]. Based on ROC, we initially selected the top three hypotheses from both pharmacophore approaches as mentioned in the above sections (Figure S1). Then, selected hypotheses were further subjected to validation by the GH approach. A test set of 106 inactive and 6 active (IC_50_ < 100) compounds were compiled and named as decoy set [30,31]. The mapping analysis revealed that in the ligand-based approach, Hypo7 was found to have the highest GH score of 0.75, followed by Hypo2 and Hypo1. On the other hand, in the structure-based approach, Hypo4 displayed a GH score of 0.83, followed by Hypo3 and Hypo1. A detailed analysis of the GH method for both approaches is shown (Table 3).

#### Hypothesis Comparison 

**Figure 4:**
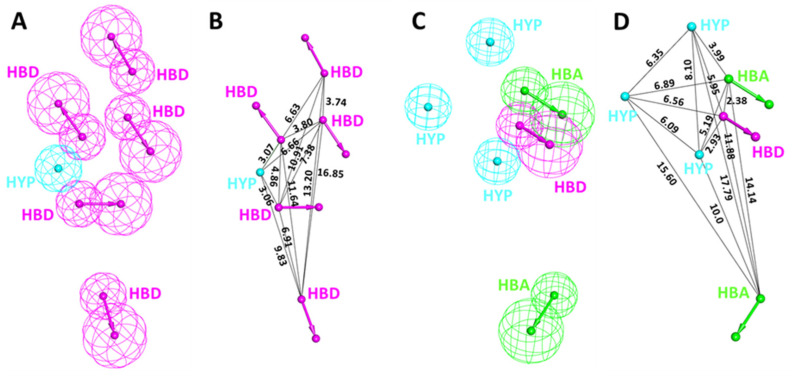
The selected pharmacophore models from the (**A**) ligand-based and (**C**) structure-based approach with the (**B**,**D**) interfeature distance between individual features of both models, respectively.

The validation results confirmed that Hypo7 (Pharm-A) and Hypo4 (Pharm-B) from ligand- and structure-based approaches, respectively, have the potential to differentiate between the active and inactive compounds of a given dataset. Therefore, these hypotheses can be further utilized for the process of virtual screening. Furthermore, the detailed inspection reveals that Pharm-A includes five HBD and one HYP feature (Figure 4A). On the other hand, Pharm-B has one HBD, two HBA, and three HYP characteristics (Figure 4C). Thus, it can be seen that the structure-based hypothesis includes more diverse features than the ligand-based hypothesis.

Interestingly, the superimposition of the Pharm-B in the active site reveals that the HBA feature situated outside of the cluster is responsible for interaction with Cys312, which is reported to provide selectivity and a covalent nature for CDK7 inhibitors (Figure 3C,D). On the contrary, this is mimicked by HBD in the case of Pharm-A. The interfeature distance was also calculated for both hypotheses, displaying the distance between individual features in Å (Figure 4B,D).

### 3.4. Drug-like Database and Virtual Screening

**Figure 5:**
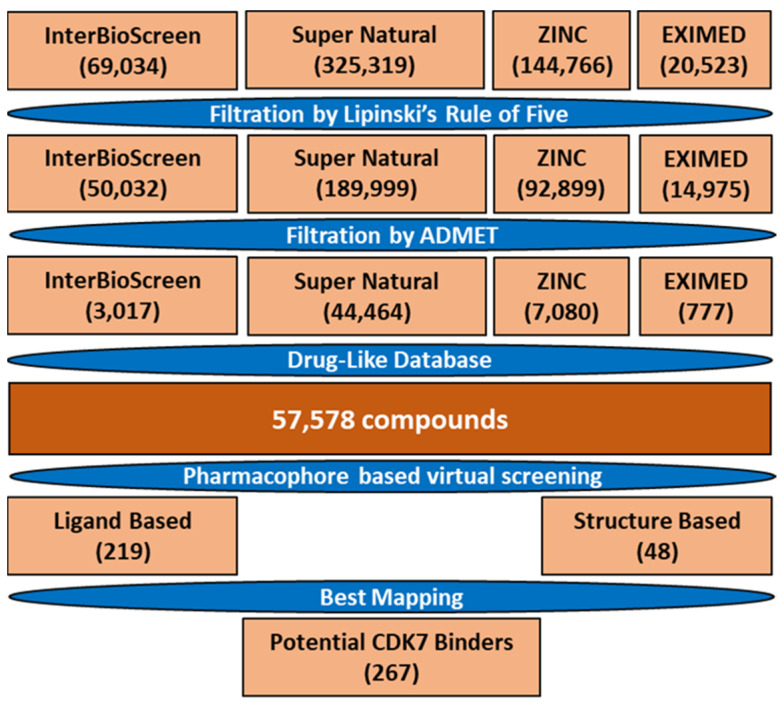
Generation and virtual screening of the natural drug-like database. Four natural compound libraries—InterBioScreen, SuperNatural2, ZINC, and ExiMed were used in the present study.

To reduce the cost and time of the screening process, we first filtered the compound libraries based on their Lipinski’s Ro5 and ADMET properties (Figure 5 and Table S3), as suggested in previous reports [43,44]. Ro5 states that compounds have drug-like characteristics when the AlogP value ≤ 5, while HBD and HBA numbers are ≤5 and 10, respectively. The molecular weight of the compounds was extended beyond Ro5 ≤ 600 Da to obtain a greater number of compounds for the screening process. In ADMET descriptors, the properties encompassing blood–brain barrier (BBB) permeability (BBBp), solubility, absorption, hepatotoxicity, and CYP2D6 were considered. The compounds were evaluated as drug-like only if they had an absorption level of 0, with solubility and BBBp level of 3. Furthermore, the compounds which predicted false value in CYP2D6 and hepatotoxicity properties were considered. The application of filters resulted in a database of 57,578 drug-like compounds (Figure 5). 

Selected pharmacophore models were then used to screen the database, and the analysis revealed that Pharm-A and Pharm-B mapped a total of 219 and 48 compounds, respectively (Figure 5). Additional manual inspection of the obtained 267 compounds resulted in 195 compounds that mapped correctly on the hypothesis. The selected compounds were then subjected to molecular docking with CDK7.

### 3.5. Molecular Docking

The THZ1-bound structure (PDB ID: 6XD3) revealed that it could target both the ATP-binding site as well as the site outside the kinase domain (Figure S2). A deeper inspection of the active site revealed hydrophobic interactions with residues Gly21, Lys41, Phe91, Leu144, Ala154, and hydrogen bonding with residues Met94, Glu95, Asp155, and Cys312 are essential for THZ1 interactions with CDK7. Before the docking experiment, the validation of the docking program GOLD was performed by re-docking the bound inhibitor THZ1. The results displayed a root mean square deviation < 2 Å between the docking pose and co-crystallized THZ1 pose, confirming that GOLD is suitable for our docking study (Figure S3). The REF inhibitor, CT7001, an ATP-competitive CDK7 inhibitor, displayed a GoldScore of 56.48, and THZ1, the first covalent CDK7 inhibitor, displayed a GoldScore of 55.80 (Tables S4 and S5). During the docking experiment, the compounds with better scores than both the REF inhibitors were selected initially. Finally, the compounds were filtered based on fundamental molecular interactions with the active site residues mentioned above. Our analysis revealed that the docked compounds obtained from Pharm-A followed a non-competitive mode for binding similar to CT7001. In contrast, compounds obtained from Pharm-B showed a competitive binding mode similar to THZ1. The GoldScores and SMILES IDs of the selected compounds are shown (Tables S4 and S5).

### 3.6. Molecular Dynamics Simulations

The docking results were further validated using MD simulations. A total of 24 CDK7 bound inhibitor complexes were simulated individually for 50 ns in the present study. For comparative analysis, REF inhibitors (CT7001 and THZ1) were also simulated under similar conditions. The stability of the simulated complexes during the MD run was scrutinized by analyzing the root mean square deviation (RMSD) and root mean square fluctuation (RMSF) plots. The binding affinity of the compounds towards CDK7 was calculated through MM-PBSA. Compounds and inhibitors were ranked according to the binding free energy (Tables S4 and S5). The compounds which failed to show stability during the simulation and desirable binding free energy (ΔG) values were removed from the analysis. Lastly, based on the stable binding mode, four molecules were selected and considered as hits against CDK7 (Table S6). It is noteworthy that Hit1 and Hit2 are obtained from the ligand-based approach, while Hit3 and Hit4 are from the structure-based approach.

#### 3.6.1. Root Mean Square Deviation and Fluctuations 

**Figure 6:**
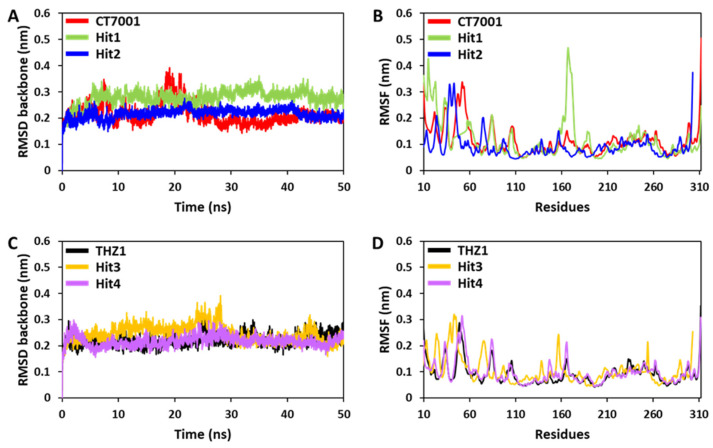
MD simulation analyses display the backbone RMSD and RMSF for (**A**,**B**) CT7001, Hit1, and Hit2, respectively, and (**C**,**D**) THZ1, Hit3, and Hit4.

The residual deviations and fluctuations were determined using backbone RMSD and RMSF analyses [67]. The backbone RMSD for CT7001, Hit1, and Hit2 demonstrated that simulated systems displayed steady-state stability after 10 ns of run time (Figure 6A). A slight bump was observed for CT7001 near 20 ns. Hit1 showed an average RMSD value of 0.27 nm, whereas a similar average value of 0.21 nm was observed for Hit2 and CT7001 (Table S6). Although the RMSD for Hit3, Hit4, and THZ1 showed stable RMSD after 5 ns, only a tiny bump was observed in the case of Hit3 near 30 ns (Figure 6C). The average RMSD value for Hit3 was 0.24 nm, whereas Hit4 and THZ1 displayed a similar value of 0.22 nm (Table S5). The RMSD values of all the simulated compounds declined after 40 ns and remained constant until the endpoint (Figure 6A,C). The RMSF is another essential parameter for the identification of the rigid and flexible region of the protein. It can be used to assess the flexibility of the backbone elements of the protein structure. The backbone RMSF was measured for all four hits and REF inhibitors (Figure 6B,D). The average RMSF value for Hit1, Hit2, Hit3, and Hit4 was 0.11, 0.09, 0.10, and 0.09 nm, respectively (Table S6). The REF inhibitors also showed similar average RMSF values of 0.09 and 0.11 for THZ1 and CT7001, respectively (Table S6). Significant fluctuations were observed for Hit1 at residues 15 and 167, Hit2 at residue 303, and CT7001 at residue 312 (Figure 6B). The observed residues are not part of the ATP-binding pocket; except these, no significant fluctuations were observed. Broadly, all simulated systems displayed <0.3 nm of RMSD and RMSF values, indicating no substantial deviations and changes, which can influence the structural stability of CDK7.

#### 3.6.2. Binding Free Energy Analysis

**Figure 7:**
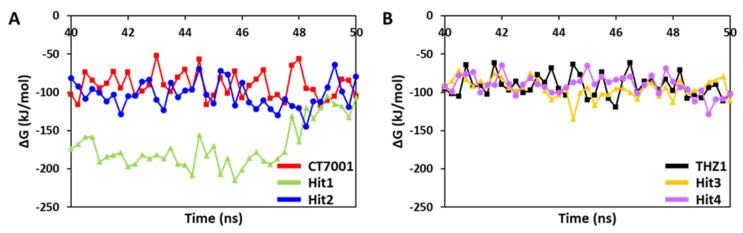
Binding free energy (ΔG) analysis for (**A**) CT7001, Hit1, and Hit2, and (**B**) THZ1, Hit3, and Hit4.

The MD simulation trajectories were used for the binding free energy (ΔG) calculations. A total of 40 snapshots were taken from the last 10 ns of stable trajectories. The REF inhibitors, CT7001 and THZ1, showed an average ΔG value of −90.58 and −91.48 kJ/mol, respectively (Figure 7 and Table 4).

The ΔG values of REF inhibitors can be considered as threshold values for the selection and ranking of hits. Our results demonstrated that a total of four compounds showed better ΔG than REF inhibitors (Table 4). The average ΔG for Hit1, Hit2, Hit3, and Hit4 was −170.01, −103.17, −94.66, and −90.59 kJ/mol, respectively. Interestingly, Hit1 displayed a significantly better binding affinity towards CDK7 than other hits and REF inhibitors. The ΔG values were further decomposed into individual components (Table 4). The decomposition showed that van der Waals interaction contributed significantly to the binding of hits with CDK7, followed by electrostatic and SASA energy. In addition, the polar solvation contributed positively to the binding and therefore opposed the complex formation.

#### 3.6.3. Binding Mode Analysis

**Figure 8:**
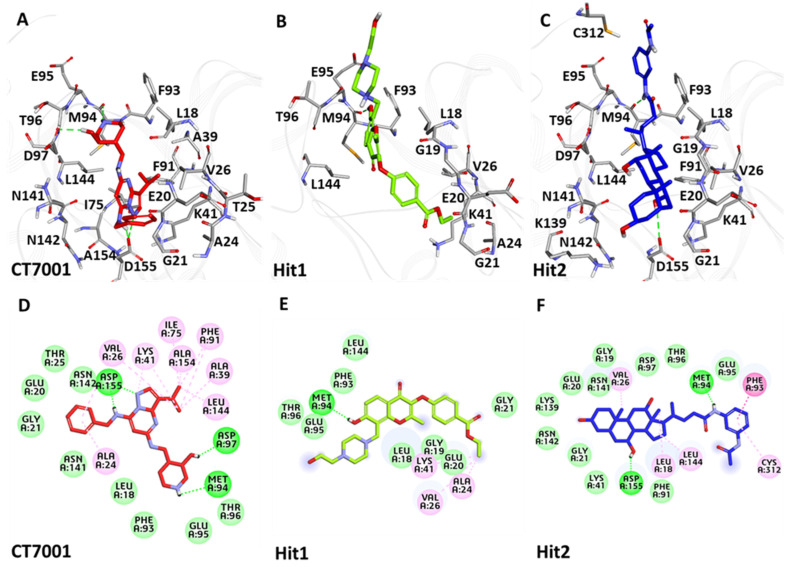
Binding mode and intermolecular interactions of CT7001 (**A**,**D**), Hit1 (**B**,**E**), and Hit2 (**C**,**F**) with CDK7. Upper and lower panel represents the interactions in 3D and 2D, respectively. The protein in the background is shown with grey lines, whereas interacting residues are shown as grey sticks. Dark green dashed lines indicate hydrogen bonds, light green: van der Waals, while the π-π and π-alkyl interactions are shown in pink.

The binding mode of the selected hits with CDK7 was evaluated using the average structure calculated from the last 10 ns of MD simulation trajectories. All the CDK7/ligand structures were superimposed in DS (Figure S4). Accordingly, the superimposition revealed that Hit1 and Hit2 occupied the ATP-binding pocket of CDK7, similar to that of CT7001 (Figure 8). A deeper insight into the molecular interactions revealed that CT7001 forms three hydrogen bonds with the ATP-binding site’s hinge region residues Met94 and Asp97, and DFG (Asp-Phe-Gly) motif residue, Asp155 (Figure 8A). The binding of CT7001 in the active site is also strengthened by van der Waals interactions with Leu18, Gly19, Glu20, Gly21, Thr25, Ile40, Asp92, Phe93, Glu95, Thr96, Asn141, Asn142, and π-alkyl interactions with Ala24, Val26, Ala39, Ile75, Phe91, Leu144, Ala154 (Figure 8D). Our Hit1 formed one hydrogen bond with hinge region residue Met94 (Figure 8B), van der Waals interactions with Leu18, Gly19, Glu20, Gly21, Thr25, Lys28, Phe93, Thr96, and Leu144, and π-alkyl interactions with Ala24, Val26, and Lys41 (Figure 8E). Hit2 forms a hydrogen bond with hinge region residue Met94 and DFG motif residue Asp155 (Figure 8C). Additionally, the van der Waals interactions with Gly19, Glu20, Gly21, Ala39, Lys41, Phe91, Glu95, Thr96, Asp97, Lys139, Asn141, Asn142, and π-alkyl interactions with Leu18, Val26, Phe93, Leu144, Cys312 were also observed (Figure 8F). Table S7 exhibits the detailed overview of H-bond distances and atoms involved in interactions with CT7001, Hit1, and Hit2. 

**Figure 9:**
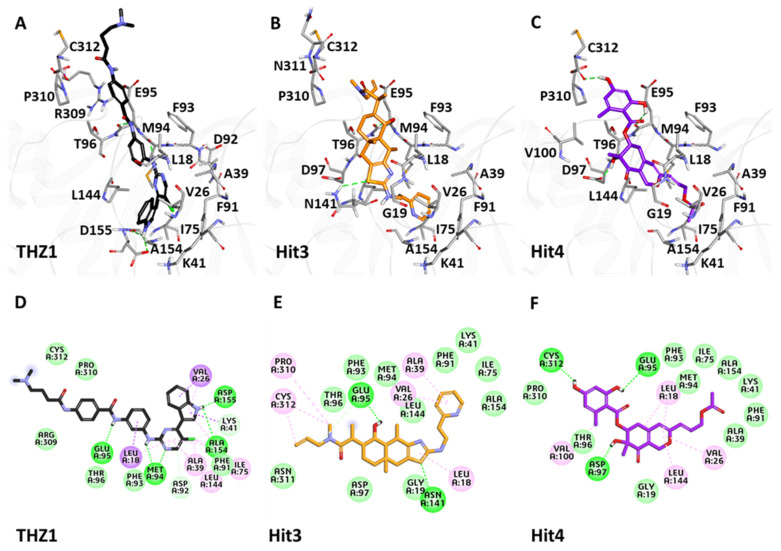
Binding mode and intermolecular interaction of THZ1 (**A**,**D**), Hit3 (**B**,**E**), and Hit4 (**C**,**F**) with CDK7. Upper and lower panel represents the interactions in 3D and 2D, respectively. The protein in the background is shown with grey lines, whereas interacting residues are shown as grey sticks. The dark green dashed lines indicating hydrogen bonds, light green: van der Waals, purple: π-sigma, while the π-π and π-alkyl interactions are shown in pink.

The superimposition of the hits inside the CDK7 active site revealed that Hit3 and Hit4 followed binding mode as seen for THZ1, indicating the hits’ covalent nature (Figure S4). Detailed inspection of molecular interactions revealed that THZ1 forms hydrogen bonds with hinge region residues Met94, Glu95, and Ala154, and DFG motif residue Asp155 (Figure 9A). Unfortunately, a hydrogen bond with Cys312 was not observed during MD simulation, which was observed in the crystal structure of CDK7 with THZ1 (PDB ID: 6XD3). THZ1 also forms van der Waals interactions with active site residues, Glu20, Gly21, Phe91, Phe93, Thr96, Arg309, Pro310, Asn311, Cys312, and alkyl interactions with Leu18, Val26, Ala39, Lys41, Ile75, Leu144 (Figure 9D). Hit3 formed hydrogen bonds with residues Glu95 and Asn141 (Figure 9B). The van der Waals interactions for Hit3 with CDK7 were observed with Gly19, Lys41, Ile75, Phe91, Phe93, Met94, Asp97, Leu144, Ala154, Asn311, whereas alkyl interactions were observed with Leu18, Val26, Ala39, Pro310, Cys312 (Figure 9E). Hit4 formed hydrogen bonds with residues Glu95, Asp97, and Cys312 (Figure 9C). Additionally, the van der Waals interactions for Hit4 with CDK7 were observed with Gly19, Ala39, Lys41, Ile75, Phe91, Phe93, Met94, Thr96, Ala154, Pro310, and π-alkyl interactions with Leu18, Val26, Val100, Leu144 (Figure 9F). The H-bond distances and atoms involved in interactions with THZ1, Hit3, and Hit4 are also shown in Table S7.

### 3.7. Specificity of Inhibitors and Hits with CDK7 over CDK2

CDK family members, CDK7 and CDK2, share highly homologous structures and have sequence identity over 44% [29]. Thus, a deeper understanding of the molecular mechanism of CDK7-specific inhibitors over CDK2 may provide essential knowledge in the structure-based drug design of isoform-selective inhibitors. To date, only a tiny number of CDK7 inhibitors have been identified with higher selectivity over CDK2 [30,31]. From the computational end, molecular docking can predict the binding mode of the potential hit compounds inside the active site of two or more isoforms. A recent study also used molecular docking methodology to explore the possible mechanism of ligand specificity in CDK family members [68]. To investigate isoform selectivity of hit compounds, we employed a similar approach with CDK2. The crystal structure of CDK2 in complex with CT7001 (PDB ID: 5JQ5) was downloaded from PDB and prepared for molecular docking, utilizing similar parameters as that for CDK7 [62]. Docking results indicated that our identified hits demonstrate lesser docking scores against CDK2 than CDK7 (Table S8). Accordingly, the representative docking pose of hit molecules with CDK2 and CDK7 were also displayed (Figure S5). It was observed that Hit1 formed a hydrogen bond with Thr14 and an unfavorable acceptor–acceptor bond with the most frequently occurring HBA feature of CDK2, Leu83 (Figure S5B). Moreover, Hit2 also exhibited unfavorable bonds with Gln131 and Leu134 of CDK2 (Figure S5C). Furthermore, hit molecules from a structure-based approach establish only hydrophobic and van der Waals interactions with CDK2 residues, whereas no hydrogen bond was observed (Figure S5E,F). Hydrogen bonds with essential CDK2 residues Ile10, Leu83, Asp86, Lys89, and Asp145 were not observed for our identified hits with CDK2 [68,69]. On the other hand, our hits display interactions with CDK7 hinge residue Met94 through hydrogen bonds as maintained during MD simulation analysis (Figure 8). Additionally, our hits target crucial CDK7 residues, Pro310 and Cys312 (Figure 9), which are not observed in CDK2 (Figure S6). These residues, along with additional CDK7 residues Val100 and Thr96 are selective for CDK7 [68]. Our docking analysis indicated interactions with the residues mentioned above via van der Waals and hydrophobic π-bonds (Table S7). While the co-crystallized CDK2 inhibitor CT7001 established hydrogen bonds with residues Leu83 and Asp145, our docking results suggested that identified hits could not follow a similar binding pattern (Figure S5). Therefore, we argue that our identified hits may be selective for CDK7 over CDK2.

### 3.8. In Silico Prediction of Pharmacokinetic Properties 

In the present study, PK properties were analyzed using the *pkCSM* webserver (Table 5). In absorption properties, the water solubility of the hits was predicted as being more soluble than the REF inhibitors, CT7001 and THZ1. According to the literature, a compound that exhibits a value >0.90 may have a high absorption rate in Caco-2 cell lines. The results indicated that CT7001 has higher permeability, whereas THZ1 has a moderate absorption rate. Interestingly, Hit2 and Hit3 also showed high absorption levels, whereas Hit4 may have a reasonable absorption level. Unfortunately, Hit1 failed to cross the Caco-2 cell line. The hits and REF inhibitors displayed an intestinal absorption rate of >30%, which indicated that all might have high intestinal absorption. The skin permeability for the compounds was found below the threshold value >−2.5, which confirmed that all compounds could easily cross the skin barriers. P-glycoprotein I, also known as multi-drug resistance protein 1 (MDR1), functions as a biological barrier by extruding toxins and xenobiotics outside of the cell. P-gp II or MDR2 functions as a phospholipid translocator. Results indicated that all the hits and REF inhibitors are P-gp substrates. The accumulation of these compounds can be reduced in specific tissues. P-gp I/II inhibition results showed that Hit2, Hit3, and THZ1 are predicted to inhibit both the variants studied. Hit1 was not predicted to inhibit P-gp II, whereas CT7001 was not predicted to inhibit P-gp I. Hit4 was not predicted to be a P-gp I/II inhibitor. In the distribution the following properties: volume of distribution (VDss), blood–brain barrier permeability (BBBp), and central nervous system (CNS) permeability (CNSp) were considered. VDss below 0.71 L/kg is considered low, and above 2.81 L/kg is considered high. The higher the VDss, more the drug is distributed in tissues. Here, CT7001 showed higher VDss, followed by Hit1, Hit3, and Hit2. Hit4 and THZ1 showed the least values of VDss. If a compound showed a value of >0.3 for BBBp and >−2 for CNSp, they can cross the BBB and CNS. Interestingly, all the hits and REF inhibitors were not predicted to be permeable for both the parameters; hence, chances of brain-related toxicities are negligible. Cytochrome P450 (CYP450) is a vital detoxification enzyme found in the liver. It oxidizes the xenobiotics to facilitate their excretion. The two main isoforms of cytochrome responsible for drug metabolism are 2D6 and 3A4, which were studied. The results indicate that none of the hits and REF inhibitors were predicted as substrates or inhibitors for the 2D6 isoform of CYP450. Unfortunately, Hit1, Hit3, and both REF inhibitors were predicted as 3A4 substrates and inhibitors. Hit2 and Hit4 were not predicted to be 3A4 inhibitors. Additionally, Hit4 was not predicted as a 3A4 substrate. Clearance can be used to calculate the rate at which drugs must be added to the circulation to maintain the steady-state plasma concentration. The clearance results showed that Hit1, CT7001, and Hit4 might have a higher clearance rate than Hit2, Hit3, and THZ1, which may have an increased half-life. The critical parameters of pharmacokinetic toxicity were also studied. None of the hits and the REF inhibitors were predicted to be mutagenic in AMES toxicity prediction. The maximum tolerated dose and oral rat toxicity properties were also predicted for hits and REF inhibitors, and are reported in our study (Table 5). The inhibition of human ether-a-go-go-related gene (hERG), which encodes a potassium ion (K) channel with two subtypes, hERG I and hERG II, was also predicted. The hERG I inhibitors may lead to cardiotoxicity-related effects. Hit compounds were not predicted to be hERG I inhibitors. hERG II is known to play a role in insulin secretion; all hits, except Hit4, were predicted to be hERG II inhibitors, and therefore may affect the glucose level. The *pkCSM* hepatotoxicity results showed that all hits and REF inhibitors might have hepatotoxic effects, but the results predicted using DS showed that hits could not be hepatotoxic. Nevertheless, these effects can be further cleared via in vitro studies. Our results showed that none of the hits and REF inhibitors were involved in skin sensitization allergic reactions.

## 4. Discussion

Cyclin-dependent kinase 7 (CDK7) regulates the cell cycle and transcription and, therefore, plays a key role in cancer development and progression [70]. The role of CDK7 has been reported in multiple human cancers and, therefore, is considered a promising therapeutic target [30]. Several investigations have found CDK7 inhibitors so far; however, first-generation inhibitors have shown considerable adverse effects, limiting their usage in clinical trials [71]. Another reason reported for the failure of first-generation inhibitors is the higher level of structural similarity among CDK family members. In the case of CDK7, it shares a 44% sequence identity with its family member, CDK2 [29]. Therefore, many of the CDK7 inhibitors have been reported to target CDK2 [32]. However, only a few inhibitors with high selectivity for CDK7 have been identified in recent years, and they are now in the early stages of clinical studies [31]. Hence, the inhibitors which can selectively inhibit CDK7 are of primary importance. Since CDK7 plays such a critical role in cell proliferation and transcription, there is a constant need for research to find effective inhibitors that can control overexpression and combat emerging cancer resistance. Therefore, we employed a ligand- and structure-based pharmacophore modeling approach complexed with a series of other computational methods as a valuable strategy for targeting CDK7 inhibition (Figure 1). The combination approach can retrieve more drug-like compounds from databases as both works on different principles [72,73]. To our knowledge, this is the first pharmacophore-based study to search for novel and selective CDK7 inhibitors. In the ligand-based approach, due to the small number of inhibitors known to date against CDK7 [31], a common feature approach was selected where a limited number of inhibitors are required as the training set (Figure 2) [43]. The only available covalent inhibitor, THZ1-bound structure (PDB ID: 6DX3), was used in the structure-based approach (Figure 3) [39]. Both approaches generated a total of ten hypotheses each (Table 1 and Table 2). The hypotheses were validated with two well-known methods- ROC and GH approach [41,42]. According to our hypothesis validation outcomes Hypo7 and Hypo4 from the ligand- and structure-based approach, respectively, can filter out known active and inactive compounds (Table 3, Figure 4). As a result, both hypotheses were used for virtual drug-like database screening. The mapping resulted in 267 drug-like compounds showing the potential to interact with CDK7 (Figure 5). The binding potential of obtained compounds was then studied by a molecular docking study [74,75]. Two well-known selective CDK7 inhibitors, CT7001 and THZ1, were used in the docking studies for comparative analysis. Recently, Wang et al., reviewed the inhibitor design studies of CDK7 and concluded that one or more hydrogen bonds with ATP-binding site residues, Gly21, Phe91, Met94, Asp155, Thr170, and Cys312, located outside the kinase domain are responsible for the inhibition [30]. Therefore, we selected only those compounds that displayed a similar binding mode as CT7001 and THZ1, greater docking scores than both REF inhibitors, and key molecular interactions with residues mentioned above. The representative pose of THZ1 and CT7001 in the docking study displayed a GoldScore value of 55.8 and 56.48, respectively. The molecular docking results further revealed that 13 compounds from the ligand-based approach and 11 from the structure-based approach have a greater docking score than CT7001 and THZ1 (Tables S4 and S5). One of the major limitations of the docking study is that they do not consider real-time dynamics of the protein–ligand interaction [47]. To study the real-time dynamics, we employed MD simulation studies. The selected protein–ligand complexes were prepared and subjected to MD simulations [43,44,59]. For comparative study, the known inhibitors THZ1 and CT7001 were also simulated. The MD study revealed that all the simulated systems showed stable RMSD and RMSF < 0.3 nm (Figure 6 and Table S6), which are the criteria generally used in the stability assessment of simulated complexes [67,76]. We were further interested in studying the binding affinity of the simulated compounds compared to known inhibitors. For this purpose, a well-known method, MM-PBSA, was employed using the *g_mmpbsa* tool [58]. The analysis revealed that CT7001 showed average binding free energy of −90.58 kJ/mol, and THZ1 displayed −91.48 kJ/mol (Figure 7, Table 5). The previous reports confirm that the lower the binding free energy values, the higher the affinity of the molecules towards the protein [43,75,76]. A similar relationship was also observed in some combined in silico and in vitro studies [77,78]. Our analysis found that Hit1 displayed significantly better binding free energy, −170.01 kJ/mol compared to REF inhibitors; this was followed by Hit2 −103.17, Hit3 94.66, and Hit4 −90.58. All the hits displayed better binding free energies than CT7001, whereas THZ1 showed slightly better binding affinity than Hit4. Although four other compounds demonstrated better binding energies than THZ1 and CT7001 (Table S5), they were not considered for further analysis because they displayed a slightly different binding mode than REF inhibitors with CDK7. The binding mode of the compounds was assessed using the average structure extracted from the last 10 ns MD simulation trajectories. CT7001 is known to inhibit CDK7 with an IC_50_ of 40 nM and CDK2 with an IC_50_ of 620 nM via an in vitro kinase assay [35]. The study by Hazel et al., demonstrated that MD simulation of CT7001/CDK7 could form hydrogen bonds with hinge region residue Met94, and G-rich loop residues Gly21, Asp137, and Asp155. Additionally, using the Asp155 mutant, it has been confirmed that interaction with Asp155 is key for the selectivity of the CT7001 towards CDK7 [62]. Our study observed that Hit1 and Hit2 obtained a similar binding mode as CT7001 (Figure 8). Hit1 forms a hydrogen bond with hinge region residue Met94; unfortunately, molecular interaction with Asp155 was not observed. Hit2 was observed to form hydrogen bond interactions with Met94 and Asp155, which enhances the selectivity towards CDK7. Interestingly, Hit2 also forms alkyl interactions outside the kinase domain with Cys312, which is reported to provide a covalent nature to THZ1. The phenylaminopyrimidine derivative THZ1 is reported as the first covalent inhibitor for CDK7 with an IC_50_ of 3.2 nM [36]. The docking studies indicated that THZ1 targets the CDK7 kinase domain and outside the domain via interacting with Cys312 through a hydrogen bond. The study also indicated that mutation of Cys312 to Ser312 prevented the THZ1 binding with CDK7 in a covalent fashion. This confirms the role of Cys312 interaction in covalent inhibition [36]. However, there is no MD simulation study reported to date for THZ1 dynamics. Here, we obtained a similar binding mode as reported, but instead of a hydrogen bond, a van der Waals interaction was observed for THZ1 with Cys312 (Figure 9). This may happen due to the position of Cys312 as it is present at the last position or maybe due to a change in dynamics, as docking studies do not consider the protein flexibility. Our analysis revealed that THZ1 forms a hydrogen bond with hinge region residues Met94, Glu95, Ala154, and Asp155. Hit3 was observed to form hydrogen bonds with Glu95 and Asn141. Unfortunately, a hydrogen bond with Cys312 was not observed in the case of Hit3 but it formed a π-alkyl interaction with the Cys312, suggesting the compound may act as a covalent inhibitor. Hit4 binding mode revealed that it forms a hydrogen bond with Glu95, Asp97, and Cys312 located outside the kinase domain, important for covalent inhibition (Figure 9). As mentioned earlier, CDK7 has similarities with CDK2; we next investigated for selectivity of the identified hits. For this purpose, a molecular docking study was performed with the CDK2 crystal structure, using similar parameters. The docking results confirmed that the docking scores were less than the co-crystal ligand CT7001 of CDK2 (Table S8). The structural details reveal that residues Lys33, Asp86, Gln131, and Asp145 are responsible for the polar interactions [68]. The hydrogen bond with Leu83 of CDK2 was reported in more than 90% of the CDK2 inhibitor interactions [68]. Our docking results followed the same interactions for the REF inhibitors CT7001 and THZ1. Interestingly, hits were not found to interact with the polar interaction site of CDK2 via hydrogen bond. Hit1 formed one hydrogen bond with Thr14, and Hit2 formed a hydrogen bond with Asn132, and both the residues are not reported to contribute much to inhibitor interactions. Additionally, our hits formed various interactions with CDK7 residues, which are not similar in CDK2. The sequence alignment of CDK7 with CDK2 revealed that ATP-binding site residues (Leu18, Ala24, Thr25, Glu95, Thr96, Val100, and Gln141) and residues outside the kinase domain (Arg309, Pro310, Asn311, and Cys312) are aligned with different CDK2 residues (Figure S6). Interestingly, our hits were found targeting these residues via various types of interactions (Table S7). This indicates that identified hits might show selectivity for CDK7 (Figure 8 and Figure 9). As part of the final assessment strategy, the detailed PK properties were assessed for REF inhibitors and hits, using the well-known tool *pkCSM* (Table 5). Results indicated that our hits showed slightly better PK properties than REF inhibitors. Among all, Hit4 was observed to be the best candidate in terms of absorption, distribution, metabolism, and toxicity parameters. Lastly, we provided the scaffolds comprised of the hits, IUPAC names, and database IDs (Table 6). Hit1 (ZINC20392430) and Hit4 (SN00262261) were observed to encompass benzoate scaffolds, while Hit2 (SN00112175) and Hit3 (SN00004718) were perceived to be pentamide and propanamide, respectively.

## 5. Conclusions

To identify new potential scaffolds against CDK7, two commonly used pharmacophore modeling approaches, ligand and structure-based, were used to generate hypotheses. The generated hypotheses were used for virtual screening of a drug-like database prepared from four natural compound databases. The filtered compounds were then subjected to molecular docking and subsequent molecular dynamics simulations for identification of their binding mode with CDK7. Additional ΔG calculations confirmed that four hits display a better binding affinity for CDK7 when compared with CT7001 and THZ1. Moreover, the selectivity of the identified hits was checked using molecular docking against CDK2, a close homolog of CDK7. Our results confirmed that the identified hits form polar and non-polar interactions with the residues unique to CDK7 (Ala24, Glu95, Thr96, Val100, Gln141, Pro310, Asn311, and Cys312), reported to enhance the selectivity. As a result, we recommend that future drug design studies focus on these residues in order to develop covalent inhibitors of CDK7, in line with earlier reports. Finally, the pharmacokinetic (PK) properties were investigated, and analyses revealed that hits have better PK properties when compared with CT7001 and THZ1. We argue that our identified hits will help to design novel drugs for CDK7.

## Figures and Tables

**Table 1:** The chemical features of 10 hypotheses that were generated for CDK7 using the *Hip-Hop* algorithm.

Sr. No.	Features ^a^	Rank ^b^	Direct Hit ^c^	Partial Hit ^d^	Max Fit ^e^
Hypo1	HYA, HBD, HYP, HBD, HBD, HBD	69.73	1111	0000	6
Hypo2	HYA, HYA, HBD, HYP, HBD, HBD, HBD	69.51	1111	0000	7
Hypo3	HYA, HBD, HYP, HBD, HBD, HBD	68.80	1111	0000	6
Hypo4	RA, HYA, HYP, HBD, HBD, HBD	68.72	1111	0000	6
Hypo5	RA, HYA, HYP, HBD, HBD, HBD	68.39	1111	0000	6
Hypo6	RA, HYA, HYP, HBD, HBD	68.39	1111	0000	6
Hypo7	HYA, HBD, HBD, HBD, HBD, HBD	68.18	1111	0000	6
Hypo8	HYA, HBD, HYP, HBD, HBD, HBD	67.86	1111	0000	6
Hypo9	HYA, HBD, HYP, HBD, HBD, HBD	67.79	1111	0000	6
Hypo10	HYA, HBD, HYP, HBD, HBD, HBD	67.71	1111	0000	6

^a^ Features: HBA—hydrogen bond acceptor, HBD—hydrogen bond donor, HYP—hydrophobic, RA—ring aromatic. ^b^ Rank: Higher the ranking score, probability of chance correlation is less. ^c^ Direct hit: Indicates whether (1 or 0) a training set molecule mapped every feature in hypothesis. ^d^ Partial hit: A training set molecule mapped every but one feature in hypothesis. ^e^ Max fit: Maximum number of features in a hypothesis.

**Table 2:** The chemical features of 10 hypotheses that were generated for CDK7, using structure-based pharmacophore modeling.

Sr. No.	Number of Features	Features Set	Selectivity Score
Hypo1	6	HBA, HBA, HBD, HYP, HYP, HYP	10.31
Hypo2	6	HBA, HBD, HYP, HYP, HYP, HYP	10.31
Hypo3	6	HBA, HBD, HYP, HYP, HYP, HYP	10.31
Hypo4	6	HBA, HBA, HBD, HYP, HYP, HYP	10.31
Hypo5	6	HBA, HBA, HBD, HYP, HYP, HYP	10.31
Hypo6	6	HBA, HBA, HBD, HYP, HYP, HYP	10.31
Hypo7	6	HBA, HBA, HYP, HYP, HYP, HYP	9.39
Hypo8	5	HBA, HBD, HYP, HYP, HYP	8.79
Hypo9	5	HBA, HBD, HYP, HYP, HYP	8.79
Hypo10	5	HBA, HBD, HYP, HYP, HYP	8.79

**Table 3:** Pharmacophore validation of ligand- and structure-based hypotheses by the Güner–Henry approach.

Sr. No.	Parameters	Ligand-Based			Structure-Based		
		Hypo1	Hypo2	Hypo7	Hypo1	Hypo3	Hypo4
1	Total number of compounds in the database (D)	110	110	110	110	110	110
2	Total number of active compounds in the database (A)	6	6	6	6	6	6
3	Total number of hits retrieved by pharmacophore model from the database (Ht)	11	8	5	3	4	2
4	Total number of active compounds in the hit list (Ha)	5	5	4	2	3	2
5	% Yield of active ((Ha/Ht) × 100)	45.45	62.5	80	66.66	75	100
6	% Ratio of actives ((Ha/A) × 100)	83.33	83.33	66.66	33.33	50	33.33
7	False negatives (A-Ha)	1	1	1	4	3	4
8	False positives (Ht-Ha)	5	3	3	1	1	0
9	Goodness of fit score (GF)	0.51	0.65	0.75	0.57	0.68	0.83

**Table 4:** The distribution of the total binding free energy scores for reference inhibitors and identified hits with CDK7 calculated through MM-PBSA methodology.

Inhibitors	van der Waals (kJ/mol)	Electrostatic (kJ/mol)	Polar Solvation (kJ/mol)	SASA Energy (kJ/mol)	Binding Energy ΔG_bind_ (kJ/mol)
Hit1	−191.19 +/− 14.45	−309.22 +/− 30.04	355.74 +/− 44.97	−25.34 +/− 1.40	−170.01 +/− 29.50
Hit2	−164.36 +/− 14.68	−47.28 +/− 20.40	128.50 +/− 26.24	−20.02 +/− 1.73	−103.17 +/− 17.65
Hit3	−167.45 +/− 10.86	−22.27 +/− 7.28	115.57 +/− 17.06	−20.50 +/− 1.07	−94.66 +/− 12.26
Hit4	−147.54 +/− 11.28	−17.71 +/− 9.15	91.10 +/− 13.54	−16.44 +/− 1.65	−90.59 +/− 12.80
THZ1	−151.40 +/− 11.25	−22.06 +/− 15.20	98.27 +/− 18.60	−16.29 +/− 1.06	−91.48 +/− 14.79
CT7001	−181.13 +/− 13.51	−44.09 +/− 16.30	154.73 +/− 31.72	−20.09 +/− 1.20	−90.58 +/− 17.08

**Table 5:** In silico prediction of ADMET properties for reference inhibitors and identified hits.

ADMET Properties		Hit 1	Hit 2	Hit 3	Hit 4	CT7001	THZ1	Unit
Absorption	Water solubility	−3.57	−4.40	−5.18	−3.70	−3.18	−3.26	log mol/L
	Caco-2 permeability	0.01	1.00	1.09	0.45	1.26	0.86	log Papp in 10^−6^ cm/s
	IA (human)	64.37	84.75	95.29	60.14	89.43	93.01	% Absorbed
	Skin permeability	−2.74	−2.73	−3.14	−2.79	−2.73	−2.73	log Kp
	P-gp substrate	Yes	Yes	Yes	Yes	Yes	Yes	Yes/No
	P-gp I inhibitor	Yes	Yes	Yes	No	No	Yes	Yes/No
	P-gp II inhibitor	No	Yes	Yes	No	Yes	Yes	Yes/No
Distribution	VDss (human)	1.49	0.04	0.50	−0.08	2.13	−0.64	log L/kg
	BBBp	−1.33	−0.67	−0.83	−1.40	−0.84	−1.26	logBB
	CNSp	−3.69	−2.12	−2.92	−3.50	−2.66	−2.2	log PS
Metabolism	CYP2D6 substrate	No	No	No	No	No	No	Yes/No
	CYP2D6 inhibitor	No	No	No	No	No	No	Yes/No
	CYP3A4 substrate	Yes	Yes	Yes	No	Yes	Yes	Yes/No
	CYP3A4 inhibitor	Yes	No	Yes	No	Yes	Yes	Yes/No
Excretion	TC	1.08	0.10	0.18	0.77	0.88	0.48	log mL/min/kg
Toxicity	AMES toxicity	No	No	No	No	No	No	Yes/No
	Max. tolerated dose (human)	0.14	−1.47	−0.3	0.32	0.15	0.43	log mg/kg/day
	hERG I inhibitor	No	No	No	No	No	No	Yes/No
	hERG II inhibitor	Yes	Yes	Yes	No	Yes	Yes	Yes/No
	Oral rat acute toxicity	2.70	3.72	2.60	3.46	2.82	2.84	LD_50_ mol/kg
	Hepatotoxicity	Yes	Yes	Yes	Yes	Yes	Yes	Yes/No
	Skin sensitization	No	No	No	No	No	No	Yes/No

Abbreviation: IA—intestinal absorption, P-gp—P-glycoprotein, VDss—volume of distribution, BBBp—blood–brain barrier permeability, CNSp—central nervous system permeability, TC—total clearance, AMES—*Salmonella typhimurium* reverse mutation assay, hERG—human ether-a-go-go-related gene.

**Table 6:** The database ID, IUPAC name, and 2D representation of the final identified hits.

Inhibitor	Database ID	IUPAC Name	2D Representation
Hit1	ZINC20392430	ethyl 4-[7-hydroxy-8-[[4-(2-hydroxyethyl)piperazin-1-ium-1-yl]methyl]-2-methyl-4-oxo-chromen-3-yl]oxybenzoate	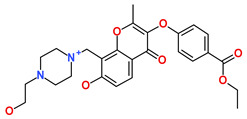
Hit2	SN00112175	(4R)-N-(3-acetamidophenyl)-4-[(3R,5S,7R,8R,9S,10S,12S,13R,14S,17R)-3,7,12-trihydroxy-10,13-dimethyl-2,3,4,5,6,7,8,9,11,12,14,15,16,17-tetradecahydro-1H-cyclopenta[a]phenanthren-17-yl]pentanamide	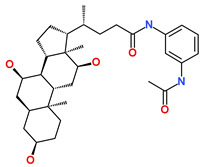
Hit3	SN00004718	(2S)-2-[(4S,4aS,5S,6S,8aS)-5-hydroxy-4,8a-dimethyl-2-[2-(2-pyridyl)ethylamino]-4a,5,6,7,8,9-hexahydro-4H-benzo[f][1,3]benzothiazol-6-yl]-N-allyl-N-methyl-propanamide	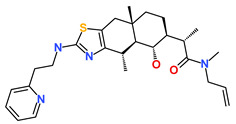
Hit4	SN00262261	[(6R,7R)-3-[(E)-3-acetoxyprop-1-enyl]-7-hydroxy-7-methyl-8-oxo-5,6-dihydro-1H-isochromen-6-yl] 2,4-dihydroxy-6-methyl-benzoate	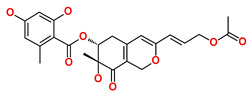

## Data Availability

Data are contained within the article.

## Supplementary Materials

The following are available online at https://www.mdpi.com/article/10.3390/biomedicines9091197/s1, Figure S1. The pharmacophore models from both the approaches obtained after ROC validation. Figure S2. Molecular docking site used in the present study. The bound ligand THZ1 was used to define the docking sphere. Figure S3. Validation of docking parameters using co-crystallized ligand THZ1 (grey) and docked pose (black). Figure S4. Binding patterns of the reference inhibitors and the hits in the active site of CDK7. Superimposition (left) of THZ1, CT7001, Hit1, Hit2, Hit3, and Hit4 (left) and its enlarged view (right). The protein is shown as grey color ribbon representation, and the ligands are shown with stick representation. Only polar hydrogen atoms are shown for clear visualization. Figure S5. The 2D molecular interactions of the reference inhibitors and identified hits with CDK2 crystal structure bound with CT7001 (PDB ID: 5JQ5). The dark green dashed lines indicating hydrogen bonds, light green: van der Waals, purple: π-sigma, red: unfavorable acceptor–acceptor, orange: π-cation, while the π-π and π-alkyl interactions are shown in pink. Figure S6. Sequence alignment of CDK7 (PDB ID: 6XD3) and CDK2 (PDB ID: 5JQ5) resulted in 44.4% identity and 67.1% similarity. The red boxes are used to show the differences between the active site and outside the active site residues (309–312) of both the proteins. The degree of identity ranges from dark cyan color (identical) to white color (non-identical). Table S1. Ligand-based pharmacophore validation using receiver operating characteristic (ROC) curve. Table S2. Structure-based pharmacophore validation using receiver operating characteristic (ROC) curve. Table S3. Details of different properties used for generation of drug-like database. Table S4. List of potential compounds obtained after molecular docking. A total of 13 compounds show a better GoldScore than reference (REF) inhibitor. The binding free energies obtained after MD simulation are also shown. Table S5. List of potential compounds obtained after molecular docking. A total of 11 compounds show better GoldScore than REF inhibitor. The binding free energies obtained after MD simulation are also shown. Table S6. Molecular docking and molecular dynamics simulation analysis of hits and reference inhibitors against CDK7. Table S7. Molecular interactions of reference inhibitors and hits with CDK7 active site residues acquired from stable molecular dynamics simulation trajectories. Table S8. The molecular docking scores of hits and reference inhibitors with CDK2 and CDK7.

## References

[1] Cui W., Aouidate A., Wang S., Yu Q., Li Y., Yuan S. (2020). Discovering Anti-Cancer Drugs via Computational Methods. Front. Pharmacol..

[2] Sanchez-Vega F., Mina M., Armenia J., Chatila W.K., Luna A., La K.C., Dimitriadoy S., Liu D.L., Kantheti H.S., Saghafinia S. (2018). Oncogenic Signaling Pathways in The Cancer Genome Atlas. Cell.

[3] Liu K., Zheng M., Lu R., Du J., Zhao Q., Li Z., Li Y., Zhang S. (2020). The role of CDC25C in cell cycle regulation and clinical cancer therapy: A systematic review. Cancer Cell Int..

[4] Lim S., Kaldis P. (2013). Cdks, cyclins and CKIs: Roles beyond cell cycle regulation. Development.

[5] Barnum K.J., O’Connell M.J. (2014). Cell Cycle Regulation by Checkpoints. Methods Mol. Biol..

[6] Malumbres M., Harlow E., Hunt T., Hunter T., Lahti J.M., Manning G., Morgan D.O., Tsai L.H., Wolgemuth D.J. (2009). Cy-clin-dependent kinases: A family portrait. Nat. Cell Biol..

[7] Galbraith M.D., Bender H., Espinosa J.M. (2019). Therapeutic targeting of transcriptional cyclin-dependent kinases. Transcription.

[8] Fisher R.P. (2005). Secrets of a double agent: CDK7 in cell-cycle control and transcription. J. Cell Sci..

[9] Tassan J.P., Jaquenoud M., Fry A.M., Frutiger S., Hughes G.J., Nigg E. (1995). In vitro assembly of a functional human CDK7-cyclin H complex requires MAT1, a novel 36 kDa RING finger protein. EMBO J..

[10] Martinez A.-M., Afshar M., Martin F., Cavadore J., Labbé J., Dorée M. (1997). Dual phosphorylation of the T-loop in cdk7: Its role in controlling cyclin H binding and CAK activity. EMBO J..

[11] Cao K., Shilatifard A. (2014). Inhibit Globally, Act Locally: CDK7 Inhibitors in Cancer Therapy. Cancer Cell.

[12] Ramanathan Y., Rajpara S.M., Reza S.M., Lees E., Shuman S., Mathews M.B., Pe’Ery T. (2001). Three RNA Polymerase II Carboxyl-terminal Domain Kinases Display Distinct Substrate Preferences. J. Biol. Chem..

[13] Glover-Cutter K., Larochelle S., Erickson B., Zhang C., Shokat K., Fisher R.P., Bentley D.L. (2009). TFIIH-Associated Cdk7 Kinase Functions in Phosphorylation of C-Terminal Domain Ser7 Residues, Promoter-Proximal Pausing, and Termination by RNA Polymerase II. Mol. Cell. Biol..

[14] Bartkova J., Zemanova M., Bartek J. (1996). Expression of CDK7/CAK in normal and tumour cells of diverse histogenesis, cell-cycle position and differentiation. Int. J. Cancer.

[15] Kim J., Cho Y.-J., Ryu J.-Y., Hwang I., Han H.D., Ahn H.J., Kim W.Y., Cho H., Chung J.-Y., Hewitt S.M. (2020). CDK7 is a reliable prognostic factor and novel therapeutic target in epithelial ovarian cancer. Gynecol. Oncol..

[16] Zhang Z., Peng H., Wang X., Yin X., Ma P., Jing Y., Cai M.-C., Liu J., Zhang M., Zhang S. (2017). Preclinical Efficacy and Molecular Mechanism of Targeting CDK7-Dependent Transcriptional Addiction in Ovarian Cancer. Mol. Cancer Ther..

[17] Greenall S.A., Lim Y.C., Mitchell C.B., Ensbey K.S., Stringer B.W., Wilding A.L., O’Neill G.M., McDonald K.L., Gough D.J., Day B.W. (2017). Cyclin-dependent kinase 7 is a therapeutic target in high-grade glioma. Oncogenesis.

[18] Lu P., Geng J., Zhang L., Wang Y., Niu N., Fang Y., Liu F., Shi J., Zhang Z.-G., Sun Y.-W. (2019). THZ1 reveals CDK7-dependent transcriptional addictions in pancreatic cancer. Oncogene.

[19] Patel H., Abduljabbar R., Lai C.-F., Periyasamy M., Harrod A., Gemma C., Steel J.H., Patel N., Busonero C., Jerjees D. (2016). Expression of CDK7, Cyclin H, and MAT1 Is Elevated in Breast Cancer and Is Prognostic in Estrogen Receptor–Positive Breast Cancer. Clin. Cancer Res..

[20] Wang Q., Li M., Zhang X., Huang H., Huang J., Ke J., Ding H., Xiao J., Shan X., Liu Q. (2016). Upregulation of CDK7 in gastric cancer cell promotes tumor cell proliferation and predicts poor prognosis. Exp. Mol. Pathol..

[21] Naseh G., Mohammadifard M., Mohammadifard M. (2016). Upregulation of cyclin-dependent kinase 7 and matrix metalloproteinase-14 expression contribute to metastatic properties of gastric cancer. IUBMB Life.

[22] Tsang F.H., Law C., Tang T.C., Cheng C.L., Chin D.W., Tam W.V., Wei L., Wong C.C.L., Ng I.O., Wong C. (2019). Aberrant Super-Enhancer Landscape in Human Hepatocellular Carcinoma. Hepatology.

[23] Jiang L., Huang R., Wu Y., Diao P., Zhang W., Li J., Li Z., Wang Y., Cheng J., Yang J. (2019). Overexpression of CDK7 is associated with unfavourable prognosis in oral squamous cell carcinoma. Pathology.

[24] Huang T., Ding X., Xu G., Chen G., Cao Y., Peng C., Shen S., Lv Y., Wang L., Zou X. (2019). CDK7 inhibitor THZ1 inhibits MCL1 synthesis and drives cholangiocarcinoma apoptosis in combination with BCL2/BCL-XL inhibitor ABT-263. Cell Death Dis..

[25] Zhou Y., Lu L., Jiang G., Chen Z., Li J., An P., Chen L., Du J., Wang H.-S. (2019). Targeting CDK7 increases the stability of Snail to promote the dissemination of colorectal cancer. Cell Death Differ..

[26] Ding L., Cao J., Lin W., Chen H., Xiong X., Ao H., Yu M., Lin J., Cui Q. (2020). The Roles of Cyclin-Dependent Kinases in Cell-Cycle Progression and Therapeutic Strategies in Human Breast Cancer. Int. J. Mol. Sci..

[27] Fisher R.P. (2018). Cdk7: A kinase at the core of transcription and in the crosshairs of cancer drug discovery. Transcription.

[28] Sava G.P., Fan H., Coombes R.C., Buluwela L., Ali S. (2020). CDK7 inhibitors as anti-cancer drugs. Cancer Metastasis Rev..

[29] Lolli G., Lowe E.D., Brown N.R., Johnson L.N. (2004). The Crystal Structure of Human CDK7 and Its Protein Recognition Properties. Structure.

[30] Wang M., Wang T., Zhang X., Wu X., Jiang S. (2020). Cyclin-dependent kinase 7 inhibitors in cancer therapy. Futur. Med. Chem..

[31] Diab S., Yu M., Wang S. (2020). CDK7 Inhibitors in Cancer Therapy: The Sweet Smell of Success?. J. Med. Chem..

[32] Sánchez-Martínez C., Lallena M.J., Sanfeliciano S.G., de Dios A. (2019). Cyclin dependent kinase (CDK) inhibitors as anticancer drugs: Recent advances (2015–2019). Bioorganic Med. Chem. Lett..

[33] Ali S., Heathcote D.A., Kroll S.H.B., Jogalekar A.S., Scheiper B., Patel H., Brackow J., Siwicka A., Fuchter M., Periyasamy M. (2009). The Development of a Selective Cyclin-Dependent Kinase Inhibitor That Shows Antitumor Activity. Cancer Res..

[34] Clark K., Ainscow E., Peall A., Thomson S., Leishman A., Elaine S., Ali S., Coombes R., Barrett A., Bahl A.K. (2017). CT7001, a Novel Orally Bio-Available CDK7 Inhibitor, Is Highly Active in in-Vitro and in-Vivo Models of AML. Blood.

[35] Patel H., Periyasamy M., Sava G., Bondke A., Slafer B.W., Kroll S.H.B., Barbazanges M., Starkey R., Ottaviani S., Harrod A. (2018). ICEC0942, an Orally Bioavailable Selective Inhibitor of CDK7 for Cancer Treatment. Mol. Cancer Ther..

[36] Kwiatkowski N., Zhang T., Rahl P.B., Abraham B.J., Reddy J., Ficarro S.B., Dastur A., Amzallag A., Ramaswamy S., Tesar B. (2014). Targeting transcription regulation in cancer with a covalent CDK7 inhibitor. Nature.

[37] Chandrasekaran B., Agrawal N., Kaushik S. (2019). Pharmacophore Development. Encyclopedia of Bioinformatics and Computational Biology: ABC of Bioinformatics.

[38] Kumar V., Kumar R., Parate S., Yoon S., Lee G., Kim D., Lee K.W. (2021). Identification of ACK1 Inhibitors as Anticancer Agents by using Computer-Aided Drug Designing. J. Mol. Struct..

[39] Greber B.J., Perez-Bertoldi J.M., Lim K., Iavarone A.T., Toso D.B., Nogales E. (2020). The cryoelectron microscopy structure of the human CDK-activating kinase. Proc. Natl. Acad. Sci. USA.

[40] Parate S., Kumar V., Hong J., Lee K. (2021). Identification of Flavonoids as Putative ROS-1 Kinase Inhibitors Using Pharmacophore Modeling for NSCLC Therapeutics. Molecules.

[41] Zou K.H., O’Malley A.J., Mauri L. (2007). Receiver-Operating Characteristic Analysis for Evaluating Diagnostic Tests and Predictive Models. Circulation.

[42] Guner O.F. (2002). History and Evolution of the Pharmacophore Concept in Computer-Aided Drug Design. Curr. Top. Med. Chem..

[43] Parate S., Kumar V., Hong J.C., Lee K.W. (2021). Computational Investigation Identified Potential Chemical Scaffolds for Hepa-ranase as Anticancer Therapeutics. Int. J. Mol. Sci..

[44] Kumar V., Parate S., Yoon S., Lee G., Lee K.W. (2021). Computational Simulations Identified Marine-Derived Natural Bioactive Compounds as Replication Inhibitors of SARS-CoV-2. Front. Microbiol..

[45] Lipinski C.A., Lombardo F., Dominy B.W., Feeney P.J. (2001). Experimental and computational approaches to estimate solubility and permeability in drug discovery and development settings. Adv. Drug Deliv. Rev..

[46] Van de Waterbeemd H., Gifford E. (2003). ADMET in silico modelling: Towards prediction paradise?. Nat. Rev. Drug Discov..

[47] Meng X.-Y., Zhang H.-X., Mezei M., Cui M. (2011). Molecular Docking: A Powerful Approach for Structure-Based Drug Discovery. Curr. Comput. Drug Des..

[48] Jones G.H., Willett P., Glen R.C., Leach A.R., Taylor R. (1997). Development and validation of a genetic algorithm for flexible docking. J. Mol. Biol..

[49] Sapundzhi F., Prodanova K., Lazarova M. (2019). Enhanced sampling in molecular dynamics. J. Chem. Phys..

[50] Pronk S., Páll S., Schulz R., Larsson P., Bjelkmar P., Apostolov R., Shirts M.R., Smith J.C., Kasson P.M., Van Der Spoel D. (2013). GROMACS 4.5: A high-throughput and highly parallel open source molecular simulation toolkit. Bioinformatics.

[51] Sapay N., Tieleman D.P. (2010). Combination of the CHARMM27 force field with united-atom lipid force fields. J. Comput. Chem..

[52] Zoete V., Cuendet M.A., Grosdidier A., Michielin O. (2011). SwissParam: A fast force field generation tool for small organic molecules. J. Comput. Chem..

[53] Bussi G., Donadio D., Parrinello M. (2007). Canonical sampling through velocity rescaling. J. Chem. Phys..

[54] Parrinello M., Rahman A. (1981). Polymorphic transitions in single crystals: A new molecular dynamics method. J. Appl. Phys..

[55] Humphrey W., Dalke A., Schulten K. (1996). VMD: Visual molecular dynamics. J. Mol. Graph..

[56] Berry M., Fielding B., Gamieldien J. (2015). Practical Considerations in Virtual Screening and Molecular Docking. Emerging Trends in Computational Biology, Bioinformatics, and Systems Biology: Algorithms and Software Tools.

[57] Genheden S., Ryde U. (2015). The MM/PBSA and MM/GBSA methods to estimate ligand-binding affinities. Expert Opin. Drug Discov..

[58] Kumari R., Kumar R., Lynn A., Open Source Drug Discovery Consortium (2014). G-mmpbsa -A GROMACS tool for high-throughput MM-PBSA calculations. J. Chem. Inf. Model..

[59] Verma A.K., Kumar V., Singh S., Goswami B.C., Camps I., Sekar A., Yoon S., Lee K.W. (2021). Repurposing potential of Ayurvedic medicinal plants derived active principles against SARS-CoV-2 associated target proteins revealed by molecular docking, molecular dynamics and MM-PBSA studies. Biomed. Pharmacother..

[60] Maddox S., Hecht D., Gustafson J.L. (2016). Enhancing the selectivity of kinase inhibitors in oncology: A chemical biology perspec-tive. Future Med. Chem..

[61] Norman R.A., Toader D., Ferguson A.D. (2012). Structural approaches to obtain kinase selectivity. Trends Pharmacol. Sci..

[62] Hazel P., Kroll S.H.B., Bondke A., Barbazanges M., Patel H., Fuchter M.J., Coombes R.C., Ali S., Barrett A.G.M., Freemont P.S. (2017). Inhibitor Selectivity for Cyclin-Dependent Kinase 7: A Structural, Thermodynamic, and Modelling Study. ChemMedChem.

[63] Han Y., Zhang J., Hu C.Q., Zhang X., Ma B., Zhang P. (2019). In silico ADME and Toxicity Prediction of Ceftazidime and Its Impurities. Front. Pharmacol..

[64] Lagorce D., Douguet D., Miteva M., Villoutreix B.O. (2017). Computational analysis of calculated physicochemical and ADMET properties of protein-protein interaction inhibitors. Sci. Rep..

[65] Pires D.E.V., Blundell T.L., Ascher D.B. (2015). pkCSM: Predicting Small-Molecule Pharmacokinetic and Toxicity Properties Using Graph-Based Signatures. J. Med. Chem..

[66] Triballeau N., Acher F., Brabet I., Pin J.-P., Bertrand H.-O. (2005). Virtual Screening Workflow Development Guided by the “Receiver Operating Characteristic” Curve Approach. Application to High-Throughput Docking on Metabotropic Glutamate Receptor Subtype 4. J. Med. Chem..

[67] Martínez L. (2015). Automatic Identification of Mobile and Rigid Substructures in Molecular Dynamics Simulations and Fractional Structural Fluctuation Analysis. PLoS ONE.

[68] Chohan T.A., Pan Y.-L., Qian H.-Y., Chen J.-Z. (2015). Molecular simulation studies on the binding selectivity of 2-anilino-4-(thiazol-5-yl)-pyrimidines in complexes with CDK2 and CDK7. Mol. BioSyst..

[69] Tripathi S.K., Muttineni R., Singh S.K. (2013). Extra precision docking, free energy calculation and molecular dynamics simulation studies of CDK2 inhibitors. J. Theor. Biol..

[70] Whittaker S., Mallinger A., Workman P., Clarke P.A. (2017). Inhibitors of cyclin-dependent kinases as cancer therapeutics. Pharmacol. Ther..

[71] Asghar U., Witkiewicz A.K., Turner N.C., Knudsen E.S. (2015). The history and future of targeting cyclin-dependent kinases in cancer therapy. Nat. Rev. Drug Discov..

[72] Arooj M., Sakkiah S.D., Kim S., Arulalapperumal V., Lee K.W. (2013). A Combination of Receptor-Based Pharmacophore Modeling & QM Techniques for Identification of Human Chymase Inhibitors. PLoS ONE.

[73] Maganti L., Grandhi P., Ghoshal N. (2016). Integration of ligand and structure based approaches for identification of novel MbtI inhibitors in Mycobacterium tuberculosis and molecular dynamics simulation studies. J. Mol. Graph. Model..

[74] Bhowmick S., AlFaris N.A., Altamimi J.Z., Alothman Z.A., Aldayel T.S., Wabaidur S.M., Islam A. (2020). Screening and analysis of bioactive food compounds for modulating the CDK2 protein for cell cycle arrest: Multi-cheminformatics approaches for anticancer therapeutics. J. Mol. Struct..

[75] Zeb A., Son M., Yoon S., Kim J.H., Park S.J., Lee K.W. (2019). Computational Simulations Identified Two Candidate Inhibitors of Cdk5/p25 to Abrogate Tau-associated Neurological Disorders. Comput. Struct. Biotechnol. J..

[76] Kumar R., Kumar V., Lee K.W. (2021). A computational drug repurposing approach in identifying the cephalosporin antibiotic and anti-hepatitis C drug derivatives for COVID-19 treatment. Comput. Biol. Med..

[77] Cheng Z.-Q., Zhu K.-K., Zhang J., Song J.-L., Muehlmann L.A., Jiang C.-S., Liu C.-L., Zhang H. (2019). Molecular-docking-guided design and synthesis of new IAA-tacrine hybrids as multifunctional AChE/BChE inhibitors. Bioorganic Chem..

[78] Silva L.R., Guimarães A.S., Nascimento J.D., Nascimento I.J.D.S., da Silva E.B., McKerrow J.H., Cardoso S.H., da Silva-Júnior E.F. (2021). Computer-aided design of 1,4-naphthoquinone-based inhibitors targeting cruzain and rhodesain cysteine proteases. Bioorganic Med. Chem..

