# Supplementary material for: Identification of CDK7 Inhibitors from Natural Sources Using Pharmacoinformatics and Molecular Dynamics Simulations
Source: Biomedicines. 2021 Sep 10;9(9):1197. doi: 10.3390/biomedicines9091197 (PMC8468199; doi:10.3390/biomedicines9091197)
Supplement: Supplementary file 1 [file biomedicines-09-01197-s001.zip › biomedicines-1316524-supplementary.pdf]

biomedicines

MDPI

(Article)

Identification of CDK7 Inhibitors from Natural Sources Using Pharmacoinformatics and Molecular Dynamics Simulations

Vikas Kumar [1,†], Shraddha Parate [2,†], Gunjan Thakur [3,†], Gihwan Lee [2], Hyeon-Su Ro [4], Yongseong Kim [5], Hong Ja Kim [6], Myeong Ok Kim [6,*] and Keun Woo Lee [1,*]

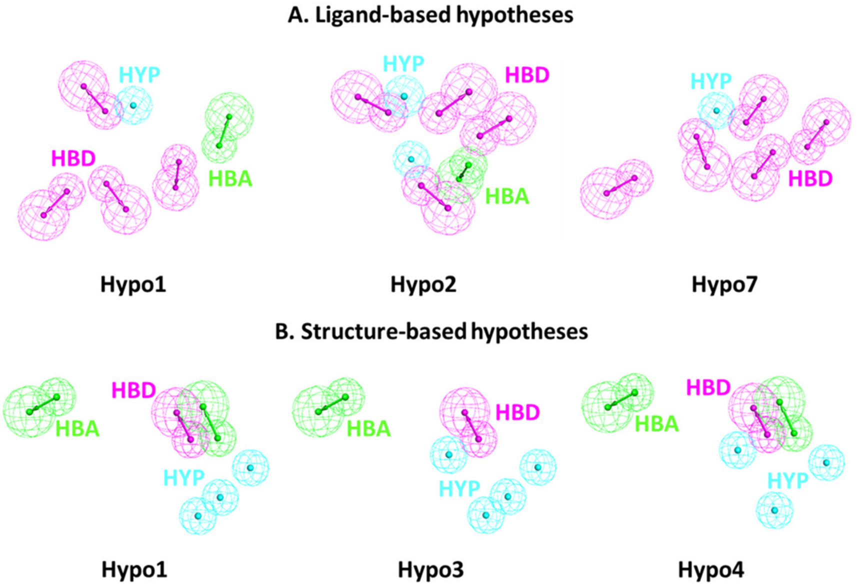

Figure S1. The pharmacophore models from both the approaches obtained after ROC validation.

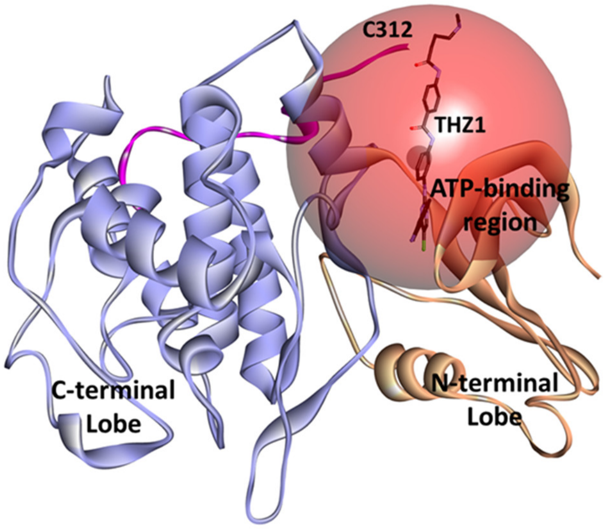

Figure S2. Molecular docking site used in the present study. The bound THZ1 was used to define the docking sphere.

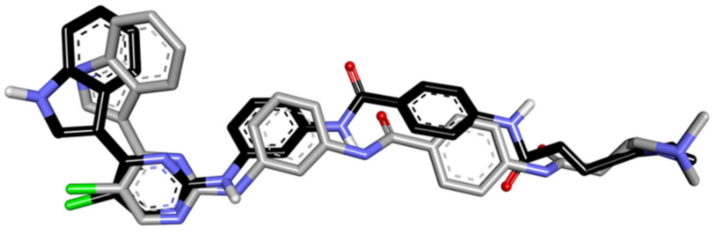

Figure S3. Validation of docking parameters using co-crystallized ligand THZ1 (grey) and docked pose (black).

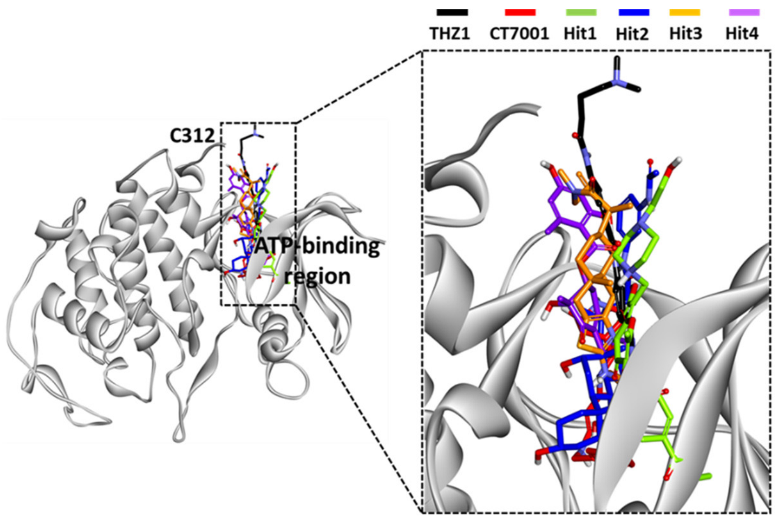

Figure S4. Binding patterns of the reference inhibitors and the hits in the active site of CDK7. Superimposition (left) of THZ1, CT7001, Hit1, Hit2, Hit3, and Hit4 (left) and its enlarged view (right). The protein is shown as grey color ribbon representation, and the ligands are shown with stick representation. Only polar hydrogen atoms are shown for clear visualization.

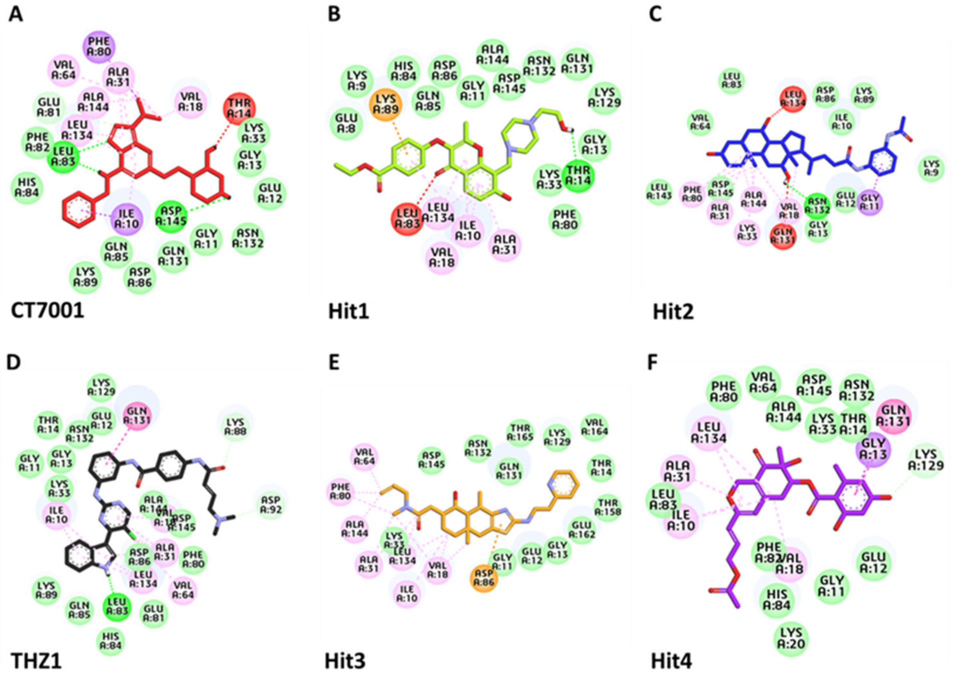

Figure S5. The 2D molecular interactions of the reference inhibitors and identified hits with CDK2 crystal structure bound with CT7001 (PDB ID: 5JQ5). The dark green dashed lines indicating hydrogen bonds, light green: van der

Waals, purple: π-sigma, red: unfavorable acceptor-acceptor, orange: π-cation, while the π-π and π-alkyl interactions are shown in pink.

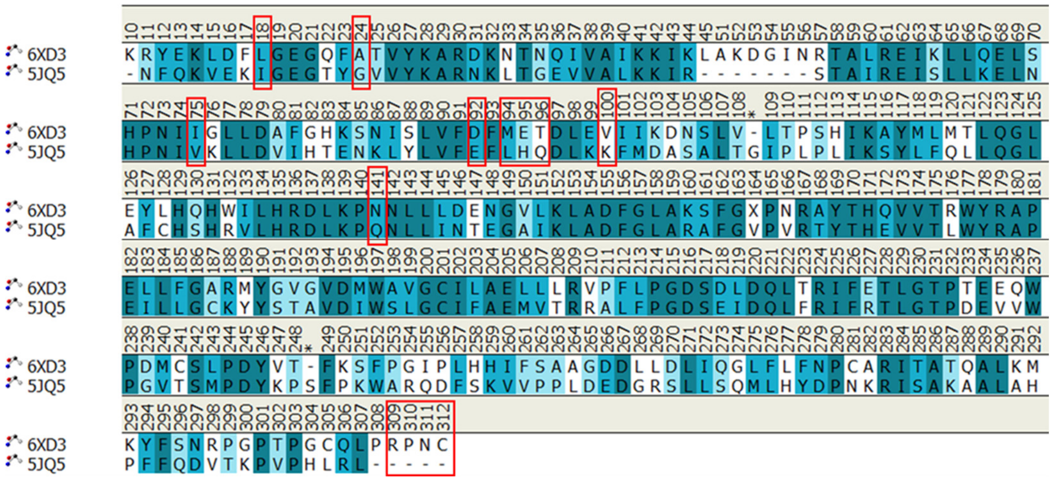

Figure S6. Sequence alignment of CDK7 (PDB ID: 6XD3) and CDK2 (PDB ID: 5JQ5) resulted in 44.4% identity and 67.1% similarity. The red boxes are used to show the differences between the active site and outside the active site residues (309-312) of both the proteins. The degree of identity ranges from dark cyan color (identical) to white color (non-identical).

Table S1. Ligand-based pharmacophore validation using Receiver Operating Characteristic (ROC) curve.

Validation with known Actives/Inactives								
**Hypothesis**	**Total actives**	**Total inactives**	**True positives**	**True negatives**	**False positives**	**False negatives**	**Sensitivity**	**Specificity**
**Hypo1**	4	8	4	6	2	0	1	0.75
**Hypo2**	4	8	4	7	1	0	1	0.87
**Hypo3**	4	8	4	6	2	0	1	0.75
**Hypo4**	4	8	4	6	2	0	1	0.75
**Hypo5**	4	8	4	6	2	0	1	0.75
**Hypo6**	4	8	4	6	2	0	1	0.75
**Hypo7**	4	8	4	7	1	0	1	0.87
**Hypo8**	4	8	4	6	2	0	1	0.75
**Hypo9**	4	8	4	6	2	0	1	0.75
**Hypo10**	4	8	4	7	1	0	1	0.70

Table S2. Structure-based pharmacophore validation using Receiver Operating Characteristic (ROC) curve.

Validation with known Actives/Inactives								
**Hypothesis**	**Total actives**	**Total inactives**	**True positives**	**True negatives**	**False positives**	**False negatives**	**Sensitivity**	**Specificity**
**Hypo1**	4	8	4	6	2	0	1	0.75
**Hypo2**	4	8	4	2	6	0	1	0.25

**Hypo3**	4	8	4	6	2	0	1	0.75
**Hypo4**	4	8	4	7	1	0	1	0.87
**Hypo5**	4	8	4	7	1	0	1	0.87
**Hypo6**	4	8	4	7	1	0	1	0.87
**Hypo7**	4	8	4	3	5	0	1	0.37
**Hypo8**	4	8	4	0	8	0	1	0
**Hypo9**	4	8	4	3	5	0	1	0.37
**Hypo10**	4	8	4	4	4	0	1	0.50

Table S3. Details of different properties used for generation of drug-like database.

**Lipinski's rule of five (Ro5)**		**ADMET descriptors**	
**Parameters**	**Cut-off**	**Parameters**	**Cut-off**
Hydrogen bond donors	≤5	Absorption level	0 (Good)
Hydrogen bond acceptors	≤10	Solubility level	3 (Good)
Molecular weight (Da)	≤600	Blood-brain barrier level	3 (Low)
AlogP value	≤5	CYP2D6 prediction	False
		Hepatotoxic prediction	False

Table S4. List of potential compounds obtained after molecular docking. A total of 13 compounds show a better Goldscore than reference (REF) inhibitor. The binding free energies obtained after MD simulation are also shown.

**Name**	**Goldscore**	**ΔG binding energy**	**SMILES IDs**
Compound 1	77.89	-51.19	OC1=CC=C(CC2NC3(C4C2C(=O)N(CCC5=CC=CC=C5)C4=O)C(=O)NC6=C3C=CC=C6)C=C1O
Compound 2	76.66	-0.742	COC1=CC(=CC=C1)\C=C2/OC3=C(C=CC(=C3)OCC(=O)NC(CC4=CC=C(O)C=C4)C(O)=O)C2=O
Compound 3	74.66	55.96	COC1=CC=C(C=C1)\C=C2/OC3=C(C=CC(=C3)OCC(=O)NC(CC4=CC=C(O)C=C4)C(O)=O)C2=O
Compound 4	72.27	-58.42	O=C(CCC1NC(=O)C2=C(NC1=O)C=CC=C2)NC3=CC=C(OCC4=CC=CC=C4)C=C3
Compound 5	72.15	4.87	CC1=CC=C(C=C1)C(=O)NC2=C(C=CC=C2)C(=O)NC(CC3=CC=C(O)C=C3)C(O)=O
Compound 6	71.07	-89.44	CC(C)C1CC(CC(=O)NCC2=CN=CC=C2)C(=CC1CNC(=O)NCCO)C
Compound 7	70.69	-47.25	CCC(C)C(CO)NC(=O)COC1=CC2=C(C=C1)C(=O)\C(O2)=C\C3=CC=C(OC)C=C3

Compound 8	68.75	-19.98	OC\C=C\C1=CC2=C(OC(CO)C(O2)C3=CC4=C(OC(C(CO)O4)C5=CC=C(O)C(=C5)O)C=C3)C=C1
Compound 9	68.22	29.59	CC(C)CC(NC(=O)N1CC(=O)NC2=C1C=CC=C2)C(=O)NC(CC3=CC=CC=C3)C(O)=O
Compound 10	66.41	76.13	OC(=O)C(CC1=CC=CC=C1)NC(=O)C(CC2=CC=CC=C2)NC(=O)N3CC(=O)NC4=C3C=CC=C4
Compound 11	65.82	-170.01	CCOC(=O)C1=CC=C(OC2=C(C)OC3=C(C[NH+]4CCN(CCO)CC4)C(=CC=C3C2=O)O)C=C1
Compound 12	65.2	63.96	CC(C)C(NC(=O)N1CC(=O)NC2=C1C=CC=C2)C(=O)NC(CC3=CC=CC=C3)C(O)=O
Compound 13	57.49	-103.17	CC(CCC(=O)NC1=CC(=CC=C1)NC(C)=O)C2CCC3C4C(O)CC5CC(O)CCC5(C)C4CC(O)C23C
CT7001 (REF)	56.48	-90.58	CC(C)C1=C2N=C(C=C(N2N=C1)NCC3=CC=CC=C3)NCC4CCNCC4O

Table S5. List of potential compounds obtained after molecular docking. A total of 11 compounds show better Goldscore than REF inhibitor. The binding free energies obtained after MD simulation are also shown.

Name	Goldscore	ΔG binding energy	SMILES IDs
Compound 1	63.56	-69.82	COC1=C(O)C=CC(=C1)CC2C(COC2=O)CC3=CC4=C(OC(C4CO)C5=CC(=C(O)C=C5)OC)C(=C3)OC
Compound 2	61.2	-118.16	COC(=O)C1=C(SC(=N1)NC(=O)CCC(C)C2CCC3C4C(O)CC5CC(O)CCC5(C)C4CC(O)C23C)C(C)C
Compound 3	60.7	-92.77	COC1=CC=C(NC(=O)CC\C(C)=C\CC2=C(O)C3=C(COC3=O)C(=C2OC)C)C(=C1)OC
Compound 4	60.07	-153.88	COCCN1CC[N+]2=C(C1)[NH]C3=C2C=CC(=C3)NC4OC(=O)C5=C4C=CC(=C5OC)OC
Compound 5	59.13	-85.22	COC1=C(O)C=CC(=C1)\C=C\C(=O)OCC(C)(O)C2=CC3=C(O2)C=C(OC)C(=C3)OC
Compound 6	58.34	-79.86	CCN(CC)C(=O)C(C)C1CCC2(C)CC3=C(N=C(NCCC4=CC=CC=N4)S3)C(C)C2C1O
Compound 7	57.39	-9.24	C\C=C(/CCC(C)(O)C1C(O)CC2C3CCC4=C(C=CC(=C4)O[S](O)(=O)=O)C3CCC12C)C(C)C
Compound 8	57.27	-62.78	CC(=O)C1=C(OC(C)(C)\C=C\C(=O)C(C)(O)C2C(O)CC3(C)C4CC=C5C(CC(O)C(=O)C5(C)C)C4(C)C(=O)CC23C)C=CC=C1
Compound 9	57.09	-90.59	CC(=O)OC\C=C\C1=CC2=C(CO1)C(=O)C(C)(O)C(C2)OC(=O)C3=C(O)C=C(O)C=C3C

Compound 10	56.8	-94.47	COC1=CC=C(OC)C2=C1[NH]C(=C2)C(=O)NCCN3CC4CC(C3)C5=[N+](C4)C(=CC=C5)[O-]
Compound 11	56.62	-94.66	CC(C1CCC2(C)CC3=C(N=C(NCCC4=CC=CC=N4)S3)C(C)C2C1O)C(=O)N(C)CC=C
THZ1 (REF)	55.8	-91.48	CN(C)CC=CC(=O)NC1=CC=C(C=C1)C(=O)NC2=CC=CC(=C2)NC3=NC=C(C(=N3)C4=CNC5=CC=CC=C54)Cl

Table S6. Molecular docking and molecular dynamics simulation analysis of hits and reference inhibitors against CDK7.

Ligands	Docking scores	RMSD (nm)	RMSF (nm)
	Goldscore	Backbone atoms	
Hit1	65.82	0.27	0.11
Hit2	57.49	0.21	0.09
Hit3	56.62	0.24	0.10
Hit4	57.09	0.22	0.09
THZ1	55.80	0.22	0.09
CT7001	56.48	0.21	0.11

Table S7. Molecular interactions of reference inhibitors and hits with CDK7 active site residues acquired from stable molecular dynamics simulation trajectories.

Hits	Hydrogen bond interactions				van der Waals interactions	π-π/π-alkyl interactions
	Amino acid	Amino acid atom	Ligand atom	Distance (<3.0 Å)		
**CT7001**	**Met94**	O	H40	2.05	Leu18, Gly19, Glu20, Gly21, Thr25, Ile40, Asp92, Phe93, Glu95, Thr96, Asn141, Asn142	Ala24, Val26, Ala39, Ile75, Phe91, Leu144, Ala154
	Asp97	OD1	H42	1.89		
	Asp155	OD1	H46	1.71		
**Hit1**	Met94	O	H47	1.94	Leu18, Gly19, Glu20, Gly21, Thr25, Lys28, Phe93, Thr96, Leu144	Ala24, Val26, Lys41
**Hit2**	Met94	O	H48	1.61	Gly19, Glu20, Gly21, Ala39, Lys41, Phe91, Glu95, Thr96, Asp97, Lys139, Asn141, Asn142	Leu18, Val26, Phe93, Leu144, Cys312
	Asp155	OD2	H83	1.61		
**THZ1**	Met94	HN	N32	2.34	Glu20, Gly21, Phe91, Phe93, Thr96, Arg309, Pro310, Asn311, Cys312	Leu18, Val26, Ala39, Lys41, Ile75, Leu144
	Glu95	O	H69	2.14		
	Ala154	O	H71	3.08		
	Asp155	OD2	H71	1.61		

**Hit3**	Glu95	O	H69	1.86	Gly19, Lys41, Ile75, Phe91, Phe93, Met94, Asp97, Leu144, Ala154, Asn311	Leu18, Val26, Ala39, Pro310, Cys312
	Asn141	HD22	S5	2.91		
**Hit4**	Glu95	O	H45	2.68	Gly19, Ala39, Lys41, Ile75, Phe91, Phe93, Met94, Thr96, Ala154, Pro310	Leu18, Val26, Val100, Leu144
	Asp97	OD1	H34	1.59		
	Cys312	OT1	H53	1.70		

Table S8. The molecular docking scores of hits and reference inhibitors with CDK2 and CDK7.

**Protein**	**Inhibitors/Hits (molecular docking scores)**					
	**CT7001**	**THZ1**	**Hit1**	**Hit2**	**Hit3**	**Hit4**
CDK2	62.68	61.02	60.19	52.15	53.83	54.01
CDK7	56.48	55.8	65.82	57.49	56.62	57.09
